# Intensive multimodal ketogenic metabolic therapy in glioblastoma: A clinical trial

**DOI:** 10.1093/noajnl/vdag165

**Published:** 2026-06-29

**Authors:** Matthew C L Phillips, Ziad Thotathil, Nur Azri Bin Haji Mohd Yasin, Charles de Groot, Alvin Tan, Marion Kuper-Hommel, Lee-Ann Creagh, Eric Ji, Nichola Naidoo, Mariska van Essen, Fouzia Ziad, Ben G Moon, Chris Frampton, Michael B Jameson

**Affiliations:** Department of Neurology, Waikato Hospital, Hamilton, New Zealand; Department of Medicine, University of Auckland, Auckland, New Zealand; Department of Radiation Oncology, Waikato Hospital, Hamilton, New Zealand; Department of Radiation Oncology, Waikato Hospital, Hamilton, New Zealand; Department of Radiation Oncology, Waikato Hospital, Hamilton, New Zealand; Department of Medical Oncology, Waikato Hospital, Hamilton, New Zealand; Department of Medical Oncology, Waikato Hospital, Hamilton, New Zealand; Department of Medical Oncology, Waikato Hospital, Hamilton, New Zealand; Department of Radiation Oncology, Wellington Hospital, Wellington, New Zealand; Department of Radiation Oncology, Wellington Hospital, Wellington, New Zealand; Clinical Trials Unit, Wellington Hospital, Wellington, New Zealand; Department of Pathology, Waikato Hospital, Hamilton, New Zealand; Midland MRI, Waikato Hospital, Hamilton, New Zealand; Department of Medicine, University of Otago, Christchurch, New Zealand; Department of Medical Oncology, Waikato Hospital, Hamilton, New Zealand; Waikato Clinical Campus, University of Auckland, Hamilton, New Zealand

**Keywords:** fasting, glioblastoma, glucose ketone index, ketogenic diet, metabolic therapy

## Abstract

**Background:**

Glioblastoma (GBM), isocitrate dehydrogenase (IDH)-wildtype, has a median overall survival of 11-14 months despite standard treatment. Ketogenic metabolic interventions that lower the glucose ketone index (GKI) may improve outcomes. We evaluated the feasibility, tolerability, and potential clinical benefit of integrating standard treatment with an intensive multimodal metabolic therapy program (MTP) in newly diagnosed IDH-wildtype GBM.

**Methods:**

Patients received standard chemoradiation and adjuvant chemotherapy alongside an MTP comprising prolonged fasting, time-restricted feeding, and a ketogenic diet. The primary outcome was the proportion sustaining a mean daily GKI ≤6 during chemoradiation. Secondary outcomes included GKI control throughout chemotherapy, body weight, body mass index, adverse events, performance, exercise, quality of life, and survival, compared with contemporary controls using unadjusted hazard ratios (HRs) and 95% confidence intervals (CIs).

**Results:**

Among 32 eligible patients, 18 commenced chemoradiation with the MTP (intention-to-treat), and 15 completed it (per-protocol). In the intention-to-treat population, 15 of 18 patients (83%) sustained a mean daily GKI ≤6 during chemoradiation. Among per-protocol patients, the GKI was 1.88 ± 0.56 during chemoradiation and 2.53 ± 0.86 throughout chemotherapy. Intentional weight loss averaged 17%, normalizing body mass index. MTP-related adverse events were mild or moderate. Exercise activity and quality of life improved. Median overall survival was 21.5 months versus 14.7 months in controls (HR = 0.42, 95% CI 0.18-0.97, *P *= .027), with 3-year survival of 27% versus 7%.

**Conclusions:**

Intensive multimodal metabolic therapy was feasible, well-tolerated, and associated with improved exercise activity, quality of life, and survival outcomes, including higher 3-year survival.

Key Points

- The MTP was feasible, well-tolerated, and had high completion rates.
- Patients sustained a low GKI, with improved exercise activity and quality of life.
- Survival was greater—median 21.5 versus 14.7 months, 3-year 27% versus 7%.

Importance of the Study

GBM (IDH-wildtype) remains almost uniformly fatal despite maximal standard treatment. This study demonstrates that combining standard chemoradiation and adjuvant chemotherapy with an intensive multimodal ketogenic MTP is feasible and well-tolerated in newly diagnosed patients. The intervention maintained a low GKI, supporting metabolic targeting of tumor growth. It was also associated with intentional weight loss, leading to a normalized mean body mass index, along with improvements in exercise activity and quality of life. Survival outcomes were promising, with a median overall survival of 21.5 months for patients receiving the MTP versus 14.7 months for those on standard treatment alone, and a 3-year survival of 27% compared with 7%. These findings provide early clinical evidence that intensive multimodal metabolic therapy may complement standard GBM treatment and support further investigation in larger controlled trials to confirm its impact on function, quality of life, and survival.

Glioblastoma (GBM), the most common primary malignant brain tumor in adults,^1^ poses major treatment challenges due to its highly invasive growth, extensive genetic heterogeneity, and resistance to immune-mediated clearance.^2^ The standard treatment protocol involves maximal surgical resection, 6 weeks of concurrent chemoradiation, and 6 cycles (5 days each) of adjuvant temozolomide chemotherapy every 28 days, historically yielding a median overall survival of 12-15 months.^2^^,^^3^ Classification based on isocitrate dehydrogenase (IDH) mutation status has further refined the GBM diagnosis, with approximately 90% of histopathological GBM cases now designated as IDH-wildtype GBM, while the remaining cases are classified as Grade 4 IDH-mutant astrocytoma.^4^ Despite receiving the same standard protocol, patients with IDH-wildtype GBM experience markedly poorer outcomes, with a median overall survival of 11-14 months, compared with 27-39 months in IDH-mutant patients.^5-7^ This pronounced survival gap highlights the pressing need to advance more effective therapeutic strategies in patients with IDH-wildtype GBM.

Cancer cells, including those in GBM, exhibit altered metabolism and impaired mitochondrial biology across various malignant tumors.^8-10^ GBM cells demonstrate markedly increased substrate-level phosphorylation through glycolytic and glutaminolytic pathways, independent of oxygen levels.^11^ They also show enhanced growth signaling through insulin, insulin‑like growth factor‑1, and mammalian target of rapamycin pathways.^12^ Additionally, GBM cells display mitochondrial abnormalities, including degenerate and uncoupled mitochondria with membranes engaged in various stages of disintegration.^13^ These mitochondria often exhibit swelling, partial or complete cristolysis, and disrupted fusion and fission processes,^14^ along with reduced respiratory chain activity, indicating deficient oxidative phosphorylation.^15^

Given these metabolic changes, integrating the standard treatment protocol with ketogenic metabolic therapy may be beneficial. This therapy encompasses the systemic metabolic and mitochondrial shifts driven by fasting and ketogenic diet regimens.^16^^,^^17^ Both approaches reduce glucose availability while boosting ketone production, facilitating a zone of “metabolic management” characterized by a lowered blood glucose-to-ketone (beta-hydroxybutyrate) ratio, known as the glucose ketone index (GKI).^18^ Beyond targeting glucose metabolism, a lowered GKI has been shown to promote differential stress resistance and sensitization, potentially enhancing the resilience of normal cells to stressors such as radiation and chemotherapy, while increasing the vulnerability of cancer cells to these treatments.^19^ In addition, a lowered GKI can suppress growth signaling, enhance immune activity, and reduce tumor-supportive inflammation.^16^ In animal studies, a GKI ≤6 correlates with smaller brain tumor volumes and better survival, which may be amplified by concurrent radiation or chemotherapy.^18^ For GBM patients, a GKI ≤2 has been proposed as “optimal” for metabolic management.^16^

Growing clinical evidence in cancer patients, including those with GBM, indicates that the tolerability and efficacy of standard treatment may be enhanced by integrating fasting or ketogenic diet protocols.^20-23^ However, this body of evidence has important limitations. First, while many studies have explored primary brain tumors, few have specifically focused on a homogenous cohort of IDH-wildtype GBM patients. Second, most research has concentrated on ketogenic diets rather than fasting regimens, and many interventions have been relatively short (typically 12 weeks or less). Third, numerous patients struggled to monitor (or sustain) a GKI ≤6 over extended periods, with only one study exploring a rigorous “multimodal” fasting and ketogenic diet protocol.^24^ However, its multiday fasts were not strategically timed with the chemoradiation or chemotherapy cycles to optimize differential stress resistance and sensitization, potentially improving survival outcomes.^25^ Finally, to date, no study has directly compared survival outcomes between newly diagnosed GBM patients receiving standard treatment with a fasting or ketogenic diet protocol versus a contemporary control group receiving standard treatment alone, highlighting a critical gap in the evidence.

On this background, we conducted a clinical trial to determine the feasibility, tolerability, and potential clinical benefit of combining standard treatment with an intensive multimodal ketogenic metabolic therapy program (MTP), which incorporated prolonged (5-day, fluid-only) fasting, time-restricted feeding (1-2 meals a day), and a modified ketogenic diet, all delivered in a precisely timed sequence, in a hospital clinic of IDH-wildtype GBM patients.

## Materials and Methods

### Trial Overview

This prospective clinical trial was registered at ClinicalTrials.gov (ID NCT04730869) on January 29, 2021, and conducted at Waikato Hospital, a tertiary hospital in Hamilton, New Zealand. Patient recruitment spanned May 2021 to July 2024, with Wellington Hospital joining as a recruitment site in January 2024. The trial received approval from the Health and Disability Ethics Committee of New Zealand and the Māori Consultation Research Review Committee at both sites. Ethical approval for data collection from eligible patients who did not participate in the MTP was granted under a broader project (approval number 2022 AM 6134).

### Patients

Potential patients were screened and recruited at the initial appointment with their radiation or medical oncologist. Eligible patients were aged 18 years or older, had a new, histologically confirmed IDH-wildtype GBM, an Eastern Cooperative Oncology Group (ECOG) performance status of 0 to 2, were planned for 6 weeks of standard chemoradiation (followed by 6 cycles of adjuvant chemotherapy), and were on a maximum dexamethasone dose of 4 mg daily at the start of the MTP. Patients were excluded if they were not eligible for 6 weeks of chemoradiation, had type 1 diabetes, or had a medical or psychiatric disorder their oncologist judged would hinder MTP completion. After assessing eligibility, the oncologist provided a brief overview of the MTP protocol and evaluated interest. Eligible, interested patients received a detailed information sheet to review at home before committing to a baseline assessment.

### Protocol

The trial protocol combined standard GBM treatment with the MTP. No additional off-label interventions or lifestyle changes were introduced during the protocol.

Standard treatment consisted of maximal surgical resection followed by concurrent chemoradiation and adjuvant chemotherapy. Chemoradiation involved radiation (60 Gy over 6 weeks) alongside daily oral temozolomide (75 mg/m^2^ of body surface area per day, 7 days per week). Chemotherapy commenced 4 weeks after the completion of chemoradiation and consisted of daily oral temozolomide (150-200 mg/m^2^ for 5 days during each 4-week cycle, with at least 6 cycles intended). Second-line treatments, such as a repeat resection, an extra 2 weeks radiation, further temozolomide, and systemic agents (bevacizumab with irinotecan or lomustine), were implemented at the discretion of the oncologist.

The MTP incorporated prolonged fasting, time-restricted feeding, and a modified ketogenic diet, applied with precise timing. Patients underwent 8 prolonged (5-day) fluid-only fasts, allowing water, salt, tea, coffee, and a magnesium supplement. The first fast began 4 days before chemoradiation, the second occurred during the third week of chemoradiation, and the remaining 6 were undertaken 4 days before each 5-day course of temozolomide. On all other days, patients followed a time-restricted ketogenic diet, consuming 1-2 meals per day, each within a 1-hour window, and fasting for the remainder of the day. Meals were self-prepared, and patients selected their meal timing, which could vary daily. By weight, meals averaged 60% fat (70%-80% by energy intake), 30% protein, 5% fiber, and 5% net carbohydrate, primarily from meats, eggs, green vegetables, nuts, seeds, and natural oils, supplemented with a daily multivitamin. Patients were advised to eat until satiated (at least 80% full). To support adherence, patients and their trial partners received a structured series of timed emails providing practical guidance and troubleshooting advice, with additional support available as needed. The MTP did not involve a nutrition specialist, as the intervention was primarily fasting-based.

After completing standard treatment, patients could choose to continue or discontinue metabolic therapy. Those who opted to continue the time-restricted ketogenic diet could obtain ketogenic meals from other sources, provided the trial neurologist approved the macronutrient ratios.

### Assessments

In the week before chemoradiation, patients and their trial partners attended a 2-h baseline assessment with the trial neurologist covering a trial overview, medical history, neurological examination, and written informed consent. Patients received an MTP booklet containing guidelines, recipes, and daily logs for bedtime blood glucose and ketone levels (see Supplementary Material), along with a blood glucose and ketone monitor (CareSens Dual, Pharmaco Diabetes). Assessments included body weight, body mass index, performance status, exercise activity, and quality of life. Performance status was measured using the ECOG scale, verified by the most recent oncologist review (scores range from 0 to 5, lower scores indicate better performance).^26^ Exercise activity was measured using the Godin Leisure-Time Exercise (GLTE) questionnaire (scores range from 0 upward, higher scores reflect greater activity).^27^ Quality of life was assessed across physical, social, emotional, functional, and brain-specific domains using the Functional Assessment of Cancer Therapy—General (FACT-G) and Brain (FACT-Br) questionnaires (FACT-G scores range from 0 to 108 and FACT-Br scores from 0 to 200, higher scores indicate better quality of life).^28^

Throughout the trial, patients underwent regular assessments by the treating oncologist and trial neurologist. In accordance with standard hospital protocol, oncology visits occurred weekly during chemoradiation, every 8 weeks throughout adjuvant chemotherapy, and every 12-16 weeks thereafter. The oncologist evaluated performance status and adverse events, graded using the National Cancer Institute Common Terminology Criteria for Adverse Events (CTCAE) version 4 (scores range from 1 to 5, higher scores indicate greater severity), and ordered an MRI scan 8-12 weeks post-chemoradiation, followed by MRI scans every 12-16 weeks. Neurology visits occurred in weeks 3 and 6 of chemoradiation, every 8 weeks thereafter until approximately 24 months, and then every 6 months. The neurologist evaluated blood glucose and ketones, body weight, body mass index, performance status (verified with the oncologist), exercise activity, quality of life, and adverse events, assessed using the CTCAE and an MTP-specific questionnaire, which asked the patient and trial partner to identify the etiology of adverse events and their level of certainty.

### Primary and Secondary Outcomes

The primary outcome was the proportion of patients achieving and sustaining functional ketosis (defined as a mean daily blood GKI ≤6) during chemoradiation (from the initial day of the first prolonged fast to 3 weeks after completion of chemoradiation). Individual and group GKIs were calculated at each time point, with the latter defined as the ratio of group mean glucose to mean beta-hydroxybutyrate, consistent with its original description.^18^ Secondary outcomes included GKI control throughout adjuvant chemotherapy (from the initial day of the cycle 1 fast to 3 weeks after the actual or intended completion of cycle 6, using the latter for patients completing fewer than 6 cycles), as well as body weight, body mass index, adverse events, performance status, exercise activity, and quality of life during the trial (from the start of chemoradiation to the actual or intended completion of chemotherapy). Progression-free survival was determined by a treatment-blinded neuroradiologist as the time from the date of initial diagnostic biopsy to first progression, based on the 2010 Response Assessment in Neuro-Oncology criteria.^29^ Overall survival was measured as the time from the date of initial biopsy to death from any cause.

### Statistical Analysis

Data on baseline characteristics were collected for all eligible patients, categorized as those who commenced chemoradiation with the MTP (intention-to-treat), those who completed chemoradiation with the MTP (per-protocol), and those who completed chemoradiation without the MTP (contemporary controls). The control group comprised all eligible patients who either declined or were not offered the MTP.

Sample size calculations considered that a proportion of 0.8 (80%) of enrolled patients sustaining a mean daily GKI ≤6 during standard chemoradiation would be clinically significant and demonstrate the feasibility of applying the MTP during GBM treatment. The proportion of patients sustaining a GKI ≤6 during chemoradiation was tested against a one-sided null hypothesis that the true proportion was 0.5 or lower. The study aimed to achieve a statistically significant result above 0.5, with an expected improvement of 0.3 (a true proportion of 0.8 or higher). Using a binomial exact test, 18 patients were required to obtain >80% power at a significance level of 0.05.

Secondary outcomes other than survival were analyzed using linear mixed-effects models within the per-protocol group, as these endpoints required sustained adherence to the MTP for meaningful biological and clinical interpretation. Patients who withdrew during chemoradiation lacked sufficient longitudinal exposure and were therefore excluded from these analyses. Survival outcomes were evaluated in all eligible patients using Kaplan–Meier estimates and log-rank tests, with unadjusted hazard ratios (HRs) and 95% confidence intervals (CIs) derived from log-rank statistics based on observed event counts. This approach enabled assessment of survival outcomes across all patient groups while emphasizing those associated with full adherence to the MTP, consistent with the analysis of other secondary endpoints. All secondary outcome tests were 2-sided with an α of 0.05, allowing for effects in either direction. Data are presented as mean ± standard deviation, except for survival outcomes, which are reported as medians.

## Results

### Patient Flow

**Figure 1.**
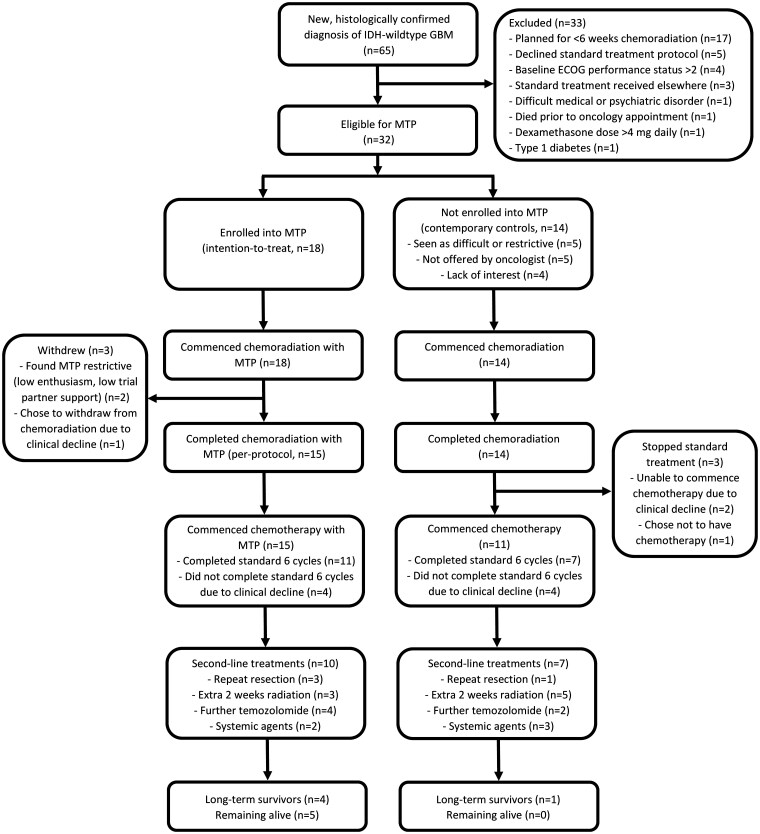
Flowchart of all patients with a new diagnosis of IDH-wildtype GBM during the 3-year recruitment period. As some patients received multiple second-line treatments, the total number of treatments exceeds the number of patients receiving them. *GBM* Glioblastoma; *IDH* Isocitrate dehydrogenase; *MTP* Metabolic therapy program.

Overall, 65 patients were diagnosed with IDH-wildtype GBM during the 3-year recruitment period, depicted in Figure 1. From this population, 32 patients (49%) met all trial eligibility criteria, among whom 18 (56%) enrolled and commenced chemoradiation with the MTP (intention-to-treat), while 14 (44%) commenced chemoradiation without the MTP (contemporary controls). Among the latter group, reasons for not enrolling included a patient perception that the MTP would be difficult or restrictive (5 patients, 36%), the MTP not being offered by the oncologist (5 patients, 36%), and lack of interest (4 patients, 29%).

Among 18 enrolled patients, 17 (94%) completed chemoradiation, and 15 (83%) completed chemoradiation with the MTP (per-protocol). Since patients were enrolled prior to initiating chemoradiation and no deaths occurred during this interval, classification did not introduce immortal time bias. The 3 patients who withdrew did so early, contributing little more than baseline data. The first patient was a 60-year-old male with an unmethylated, totally resected tumor who found the MTP restrictive in the setting of low initial enthusiasm and withdrew in week 3 (but continued chemoradiation). The second was a 57-year-old female with an unmethylated, inoperable tumor who required escalating dexamethasone (8 mg daily post-biopsy, reduced to 4 mg daily the week before chemoradiation, and then increased to 12 mg daily during treatment). Due to clinical decline, she withdrew from chemoradiation in week 3 (but continued a low-carbohydrate diet). The third was a 60-year-old female with a methylated, totally resected tumor who found the MTP restrictive in the setting of low trial partner support and withdrew in week 5 (but continued chemoradiation). All 15 per-protocol patients continued the MTP throughout chemotherapy, of whom 11 (73%) completed the standard 6 cycles. After chemotherapy, all 15 patients continued time-restricted feeding, with 12 (80%) maintaining MTP or approved ketogenic meals, while 3 (20%) adopted low-carbohydrate diets.

The per-protocol and control populations received a comparable number of second-line treatments. In the per-protocol group, 10 of 15 patients (67%) received second-line treatment, accounting for 12 treatments in total. In the control group, 7 of 14 patients (50%) received second-line treatment, accounting for 11 treatments in total.

### Baseline Characteristics

Table 1 outlines baseline characteristics for patients who commenced chemoradiation with the MTP (intention-to-treat), completed chemoradiation with the MTP (per-protocol), and completed chemoradiation without the MTP (contemporary controls). There were no significant differences in baseline characteristics between groups, although small sample size limited power to detect modest differences.

**Table 1.** Baseline characteristics for patients who commenced chemoradiation with the MTP (intention-to-treat, *n* = 18), completed chemoradiation with the MTP (per-protocol, *n* = 15), and completed chemoradiation without the MTP (contemporary controls, *n* = 14). Due to rounding, percentages may not total exactly 100%

Characteristic	Chemoradiation with MTP (intention-to-treat, *n* = 18)	Chemoradiation with MTP (per-protocol, *n* = 15)	Chemoradiation without MTP (controls, *n* = 14)
Age (years)	56.3 ± 12.3 (range, 25-73)	55.8 ± 13.4 (range, 25-73)	63.9 ± 9.1 (range, 47-80)
Gender (male)	11 (61%)	10 (67%)	6 (43%)
Ethnicity			
European	17 (94%)	14 (93%)	13 (93%)
Māori	1 (6%)	1 (7%)	0
Asian	0	0	1 (7%)
Comorbid disorders			
Previous cancer diagnosis	4 (22%)	4 (27%)	2 (14%)
Previous myocardial infarction	2 (11%)	1 (7%)	0
Prediabetes	2 (11%)	1 (7%)	0
Hypertension	4 (22%)	4 (27%)	3 (21%)
Dyslipidemia	3 (17%)	2 (13%)	1 (7%)
Chronic or paroxysmal atrial fibrillation	2 (11%)	1 (7%)	1 (7%)
Lynch syndrome	1 (6%)	1 (7%)	0
ECOG performance status at screening visit			
0 (KPS 90 or 100)	8 (44%)	8 (53%)	5 (36%)
1 (KPS 70 or 80)	8 (44%)	5 (33%)	7 (50%)
2 (KPS 50 or 60)	2 (11%)	2 (13%)	2 (14%)
MGMT promoter methylation status			
Unmethylated	14 (78%)	12 (80%)	10 (71%)
Methylated	4 (22%)	3 (20%)	4 (29%)
Tumor location			
Frontal lobe	4 (22%)	4 (27%)	2 (14%)
Temporal lobe	2 (11%)	1 (7%)	7 (50%)
Parietal lobe	4 (22%)	3 (20%)	3 (21%)
Occipital lobe	2 (11%)	2 (13%)	1 (7%)
Multiple lobes	6 (33%)	5 (33%)	1 (7%)
Tumor surgery			
Biopsy only (inoperable)	5 (28%)	4 (27%)	2 (14%)
Partial or subtotal resection	10 (56%)	10 (67%)	9 (64%)
Total resection	3 (17%)	1 (7%)	3 (21%)
Days from biopsy to chemoradiation	41.4 ± 7.7	41.9 ± 7.6	38.9 ± 7.2
Dexamethasone			
Daily pre-chemoradiation dose (mg)	0.47 ± 1.09	0.30 ± 0.70	0.71 ± 2.16
Mean daily chemoradiation dose (mg)	1.14 ± 2.25	0.57 ± 1.10	1.25 ± 1.43

*ECOG* Eastern Cooperative Oncology Group; *KPS* Karnofsky Performance Status; *MTP* Metabolic therapy program; *MGMT* O‐6‐methylguanine‐DNA methyltransferase.

### Feasibility

**Figure 2.**
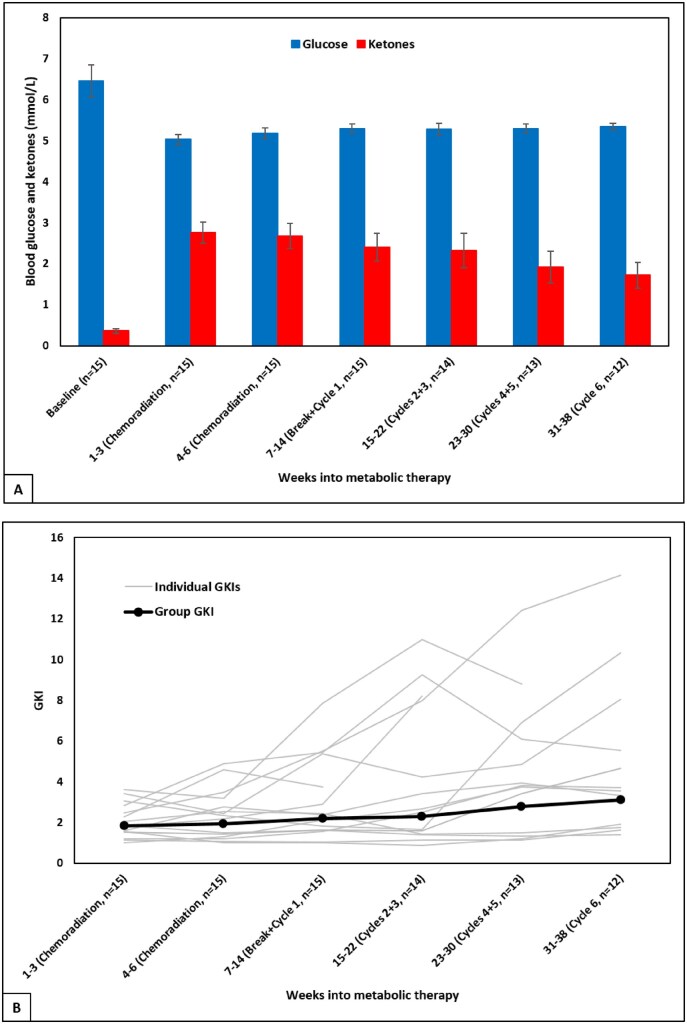

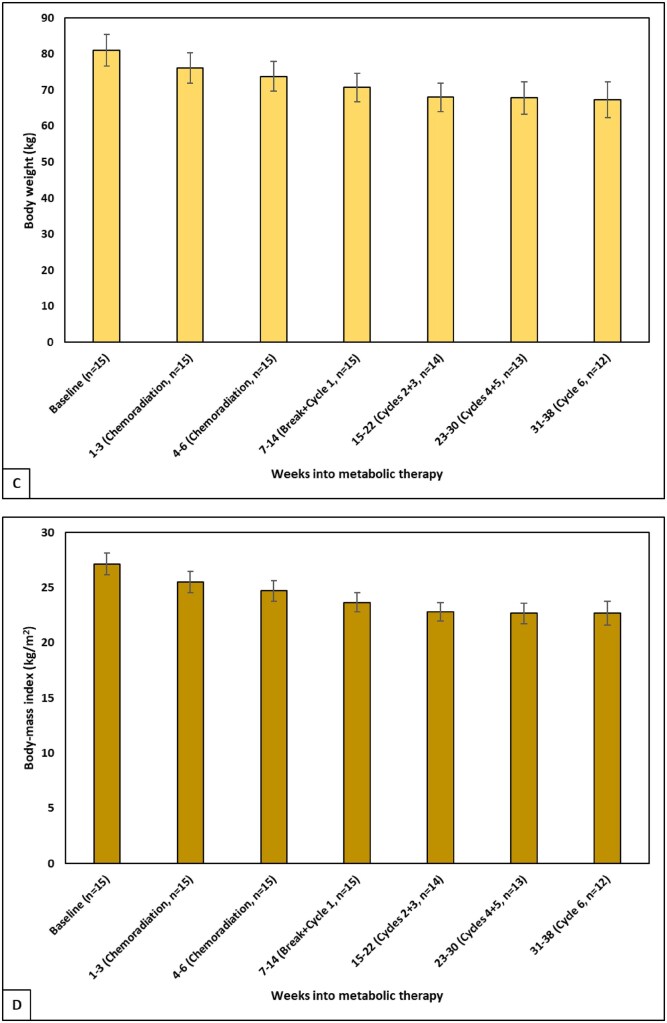
Mean daily (A) blood glucose and ketone (beta-hydroxybutyrate) levels, (B) GKIs, (C) body weights, and (D) body mass indices during the trial protocol in patients who completed chemoradiation with the MTP (per-protocol, *n* = 15). Among the 18 patients who commenced chemoradiation with the MTP, these 15 (83%) achieved the primary outcome by sustaining a GKI ≤6 during chemoradiation. All patients completed every assessment, with no data recorded for patients receiving palliative care (number of patients at each time point is shown on the x-axis). Error bars indicate standard error (removed in Panel B for clarity). *GKI* Glucose ketone index; *MTP* Metabolic therapy program.

Figure 2 illustrates mean daily blood glucose and ketone (beta-hydroxybutyrate) levels, GKIs, body weights, and body mass indices during the trial protocol in patients who completed chemoradiation with the MTP (per-protocol). Among the 18 intention-to-treat patients who commenced chemoradiation with the MTP, 15 (83%) achieved the primary outcome by sustaining a GKI ≤6 during chemoradiation, and of these 15 patients, 11 (73%) did so during chemotherapy. In the 15 per-protocol patients, the GKI fell from a baseline of 17.9 ± 12.8 (measured the evening pre-MTP) to 1.88 ± 0.56 during chemoradiation and 2.53 ± 0.86 throughout chemotherapy. Over 24 months, the GKI averaged 2.82 ± 0.71. Body weight decreased by 17% during the trial protocol (81.1 ± 17.2 to 67.4 ± 17.1 kg, *P *< .001), with a corresponding reduction in body mass index (27.1 ± 3.9 to 22.6 ± 3.4 kg/m^2^, *P *< .001); weight loss exceeded 20% in 4 of 15 patients (27%).

### Tolerability

Table 2 summarizes adverse events occurring at any time during the trial protocol among patients who completed chemoradiation with the MTP (per-protocol), excluding weight loss. In total, 74 patient-level adverse events were attributed to standard treatment (most commonly lymphopenia, fatigue, constipation, and nausea), 37 to the MTP (most commonly diarrhea, dysgeusia, dizziness, and irritability), and 74 were attributed to neither treatment or were of uncertain attribution. Most events were Grade 1 or 2, except for 8 serious events (5 Grade 3, 3 Grade 4) occurring in 5 patients, with 3 patients requiring hospitalization. The first patient was admitted with Grade 4 thrombocytopenia and Grade 3 neutropenia (both attributed to standard treatment). The second had 3 admissions for recurrent Grade 4 hyponatremia in the context of severe multifocal tumor burden with brainstem mass effect (attribution uncertain), including an episode of Grade 4 lymphopenia (attributed to standard treatment) and another episode of Grade 3 vomiting (attribution uncertain). The third was admitted with Grade 3 dizziness (attribution uncertain). The fourth and fifth patients experienced prolonged Grade 3 lymphopenia (both attributed to standard treatment). No serious adverse events were attributed to the MTP.

**Table 2.** Adverse events occurring at any time during the trial protocol among patients who completed chemoradiation with the MTP, summarized by maximum grade per patient (per-protocol, *n* = 15). Events were identified by the oncologist, patient, or trial partner and assigned attribution (possible, probable, or definite) to standard treatment (chemoradiation, adjuvant chemotherapy) or the MTP (prolonged fasting, time-restricted ketogenic diet). Each event was counted once per patient using the maximum grade; if multiple events of the same grade occurred, attribution was assigned based on the episode deemed most treatment-related. Serious adverse events are indicated and described in the text. All patients completed every assessment, with no data recorded for patients receiving palliative care.

CTCAE adverse event	Attribution (possible, probable, or definite likelihood)			Total
	Standard treatment	MTP	Neither or uncertain	
Fatigue	9	1	4	14
Diarrhea	0	9	4	13
Low lymphocytes	12 (2 Grade 3, 1 Grade 4)	0	0	12
Constipation	8	3	1	12
Nausea	8	1	3	12
Headache	5	1	6	12
Dizziness	2	4	3 (1 Grade 3)	9
Irritability	3	4	1	8
Insomnia	2	0	6	8
Pain in extremity	0	2	6	8
Alopecia	7	0	0	7
Low neutrophils	6 (1 Grade 3)	0	0	6
Dysgeusia	0	6	0	6
Myalgia	0	1	5	6
Low platelets	5 (1 Grade 4)	0	0	5
Hyponatremia	0	0	5 (1 Grade 4)	5
Seizure	0	0	5	5
Hypokalemia	0	0	5	5
Dyspepsia	0	3	1	4
Palpitations	0	1	3	4
Raised creatinine	0	0	4	4
Depression	1	0	2	3
Dysphasia	0	0	3	3
Dry skin	2	0	0	2
Anxiety	0	0	2	2
Raised liver functions	0	0	2	2
Hypoglycemia	1	0	0	1
Eyelid disorder	1	0	0	1
Urine incontinence	1	0	0	1
Anemia	1	0	0	1
Dehydration	0	1	0	1
Vomiting	0	0	1 (Grade 3)	1
Fall	0	0	1	1
Rash (shingles)	0	0	1	1
Total	74	37	74	185

*CTCAE* Common Terminology Criteria for Adverse Events; *MTP* Metabolic therapy program.

### Clinical Benefit

**Figure 3.**
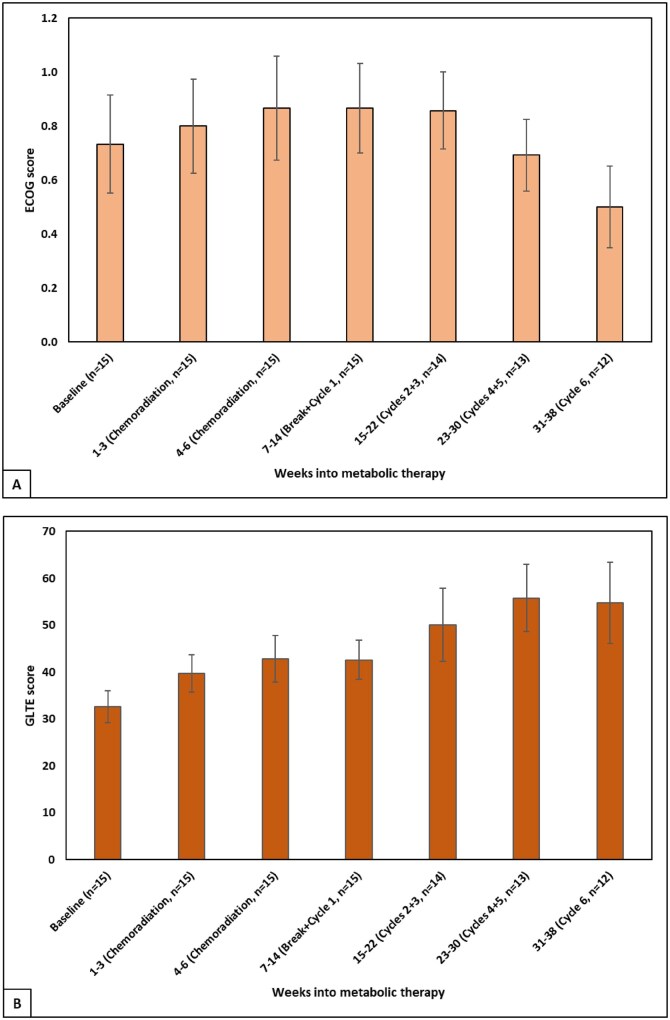

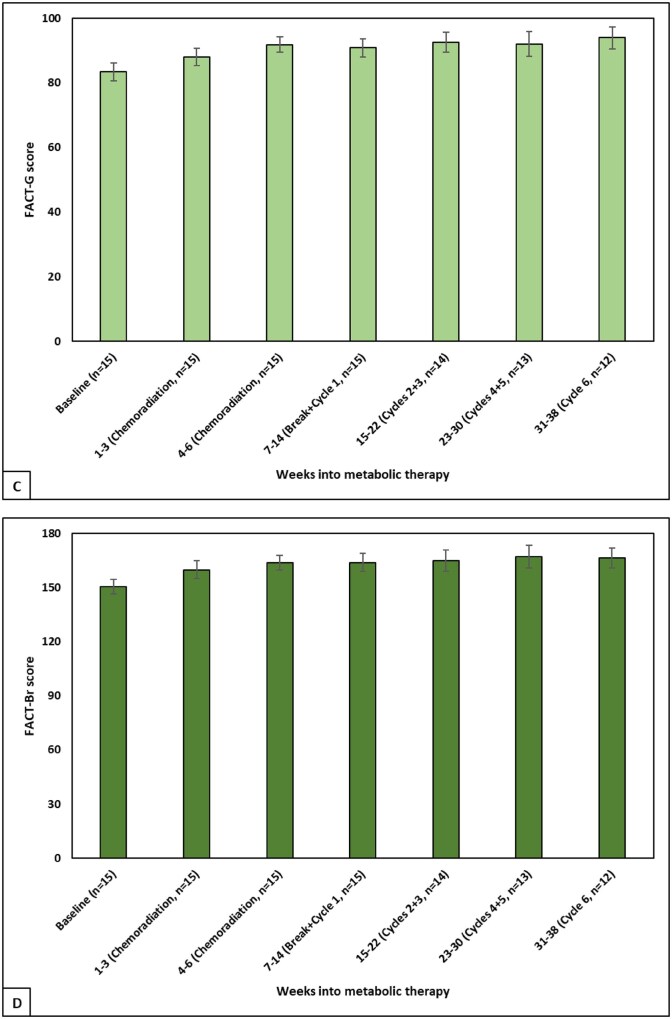
Mean (A) performance status, (B) exercise activity, (C) general quality of life, and (D) brain-specific quality of life during the trial protocol in patients who completed chemoradiation with the MTP (per-protocol, *n* = 15). All patients completed every assessment, with no data recorded for patients receiving palliative care (number of patients at each time point is shown on the x-axis). Error bars indicate standard error. *ECOG* Eastern Cooperative Oncology Group; *FACT-Br* Functional Assessment of Cancer Therapy—Brain; *FACT-G* Functional Assessment of Cancer Therapy—General; *GLTE* Godin Leisure Time Exercise; *MTP* Metabolic therapy program.

Figure 3 displays mean performance status, exercise activity, general quality of life, and brain-specific quality of life during the trial protocol in patients who completed chemoradiation with the MTP (per-protocol). The ECOG score remained stable (0.73 ± 0.70 to 0.50 ± 0.52, *P *= .39), while significant improvements were observed in the GLTE (32.7 ± 13.2 to 54.8 ± 30.0, *P *= .001), FACT-G (83.4 ± 10.6 to 93.9 ± 11.6, *P *= .007), and FACT-Br (150.5 ± 15.5 to 166.3 ± 18.9, *P *= .024) scores.

**Figure 4.**
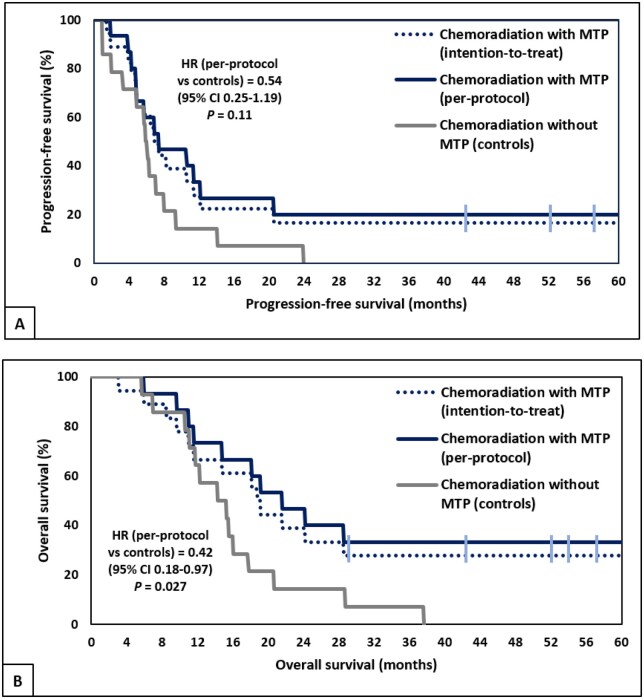
Kaplan–Meier plots showing (A) progression-free survival and (B) overall survival in patients who commenced chemoradiation with the MTP (intention-to-treat, *n* = 18), completed chemoradiation with the MTP (per-protocol, *n* = 15), and completed chemoradiation without the MTP (contemporary controls, *n* = 14). Complete survival data were obtained for all groups, with tick marks indicating censored observations for patients who remained event-free. *CI* Confidence interval; *HR* Hazard ratio; *MTP* Metabolic therapy program.

Figure 4 presents survival outcomes in patients who commenced chemoradiation with the MTP (intention-to-treat), completed chemoradiation with the MTP (per-protocol), and completed chemoradiation without the MTP (contemporary controls). Complete survival data were obtained for all groups, with observations censored in patients who remained event-free. Progression-free survival did not differ between patients completing chemoradiation with the MTP and controls (median 7.3 vs 5.9 months; HR = 0.54, 95% CI 0.25-1.19, *P *= .11). Overall survival, however, was longer in patients completing chemoradiation with the MTP (median 21.5 vs 14.7 months; HR = 0.42, 95% CI 0.18-0.97, *P *= .027). Survival differences were not evident at 12 months post-diagnosis (73% vs 64% alive), but became apparent at 18 months (67% vs 21%), 24 months (47% vs 14%), and 36 months (27% vs 7%). At the time of analysis, 4 of 15 patients (27%) had survived past 36 months (3 patients without imaging evidence of tumor), with 5 patients still alive, compared with 1 of 14 control patients (7%) surviving past 36 months, and none remaining alive. In the intention-to-treat group (all 18 enrolled patients), median overall survival was slightly lower (18.9 months), and did not reach statistical significance compared with controls (HR = 0.51, 95% CI 0.23-1.13, *P *= .073).

Overall, 4 of the 15 patients who completed chemoradiation with the MTP (27%) died within 12 months, all of whom had unmethylated GBM and high-risk features. The first patient was a 54-year-old male with an inoperable tumor; MRI demonstrated 4 discrete enhancing lesions. He declined during chemotherapy, and opted for assisted dying at 5.9 months. The second was a 24-year-old male with Lynch syndrome and a partially resected tumor; postoperative MRI showed ventricular puncture. He subsequently developed leptomeningeal dissemination and died at 9.6 months. The third, a 56-year-old male with a partially resected tumor, also had ventricular puncture on postoperative MRI. He too developed leptomeningeal dissemination and died at 10.9 months. The fourth was a 40-year-old female with a partially resected tumor; postoperative MRI demonstrated 5 residual discrete enhancing lesions. This was the same patient who experienced recurrent Grade 4 hyponatremia. She developed brainstem mass effect, required a mean dexamethasone dose of 3.95 mg daily during chemoradiation, and died at 11.5 months. Collectively, these 4 patients exhibited a higher mean daily GKI during chemoradiation compared with the remaining 11 patients (2.90 ± 0.61 vs 1.65 ± 0.44), and included the 3 highest individual GKIs observed in the trial.

## Discussion

This clinical trial evaluated one of the most intensive ketogenic metabolic interventions ever applied to IDH-wildtype GBM. Retention and adherence were high, with 15 of 18 enrolled patients (83%) achieving the primary outcome. Among these patients, the mean daily GKI was 1.88 during chemoradiation and 2.53 throughout adjuvant chemotherapy. Body weight decreased by 17%, yielding a normalized body mass index of 22.6 kg/m^2^, and MTP-related adverse events were mild or moderate. Patients completing chemoradiation with the MTP showed improved exercise activity and quality of life, with a median overall survival of 21.5 months versus 14.7 months in contemporary controls, and 3-year survival reached 27% compared with 7%.

Among 32 eligible patients, 14 (44%) did not enroll in the MTP, primarily for 2 reasons. First, 5 patients declined the MTP, citing concerns that the prolonged fasting or ketogenic diet would be difficult or restrictive. Despite their terminal diagnosis, most chose to maintain their usual dietary habits rather than undertake a potentially demanding regimen. Second, although the trial was actively promoted, 5 patients were not offered the MTP due to oversights in the referral process. Some of these patients later expressed interest but were no longer eligible after starting standard treatment. These recruitment challenges, similar to those encountered in previous GBM studies,^21^^,^^30^ highlight the need to communicate the scientific rationale for metabolic therapy more effectively to both patients and clinicians.

Retention in the MTP was strong, with 15 of 18 patients (83%) completing chemoradiation. Regarding the withdrawals, 1 patient with an inoperable, unstable GBM withdrew from chemoradiation while on high-dose dexamethasone (8-12 mg daily), which poses metabolic challenges by raising blood glucose levels and potentially impacting GBM survival,^31^^,^^32^ whereas the other 2 patients withdrew from the MTP, the first of whom lacked enthusiasm and joined at the behest of his trial partner, while the other was initially enthusiastic but lacked support from her trial partner. These experiences underscore the importance of patient motivation and trial partner support in sustaining adherence to metabolic therapy in GBM. Of the 15 patients who commenced chemotherapy, 11 (73%) completed the standard 6 cycles, exceeding both contemporary controls (50%) and historical reports for standard treatment alone (47%).^3^ This may indicate improved tolerability, consistent with preclinical and clinical evidence.^19^

Adherence to the MTP was high, with all 15 patients completing chemoradiation achieving the primary outcome, sustaining a mean daily GKI of 1.88 during chemoradiation, 2.53 throughout chemotherapy, and 2.82 over 24 months of metabolic therapy. The GKI, an objective and standardized biomarker of adherence to metabolic therapy, provides a more reliable measure than self-reported dietary compliance and correlates with treatment efficacy.^16^^,^^18^ In this trial, the prolonged fasts were designed to maximally reduce circulating fermentable fuel levels, including glucose (30%-40% reduction over 5 days),^33^ and, to a lesser extent, glutamine (15%-20% reduction).^34^ This protocol enabled patients to maintain low GKI values despite concurrent use of dexamethasone, a drug known to raise blood glucose levels,^31^ suggesting that the intensive fasting component may have helped mitigate steroid-induced hyperglycemia while requiring only a low mean daily dose of 0.57 mg during chemoradiation. Lowering the GKI also promotes differential stress resistance and sensitization, which we aimed to optimize using a modified “press-pulse” strategy.^35^ Specifically, the prolonged fasts were timed to deliver an acute, high-intensity stress to cancer cell metabolism at the onset of chemoradiation and each chemotherapy cycle (the “pulse”), while the time-restricted ketogenic diet provided ongoing, low-intensity metabolic stress (the “press”). Combined with other pleiotropic mechanisms, the low GKI achieved may have reflected a near-optimal zone of metabolic management for GBM.

During the trial, patients lost a mean 17% of body weight, with body mass index decreasing from an overweight 27.1 kg/m^2^ to a normalized 22.6 kg/m^2^. Of note, 4 of 15 patients (27%) lost over 20% of their body weight, classified as a serious adverse event by the CTCAE. In cancer, weight loss is typically considered adverse, as it is unintentional and associated with cachexia, functional decline, and increased nutritional support.^36^ However, intentional weight loss within a structured metabolic intervention may warrant a different perspective. Prior research suggests that deliberate weight loss reflects significant bioenergetic stress from ketone production, which cancer cells struggle to metabolize effectively,^10^ and may help preserve lean body mass.^37^ Intentional weight loss is also linked to lower cancer risk,^38^ and, in some contexts, improved survival outcomes in advanced cancer, including GBM.^39^^,^^40^ Moreover, in this trial, all 4 patients who lost over 20% of body weight survived past 24 months, with 3 remaining alive. Altogether, these findings raise the possibility that weight loss during metabolic therapy may be associated with beneficial outcomes, rather than being purely adverse.

Twice as many adverse events were attributed to standard treatment compared with the MTP (74 vs 37 events). Consistent with prior studies,^3^ lymphopenia and fatigue were common with standard chemoradiation and chemotherapy, whereas the prolonged fasting component of the MTP most frequently resulted in mild, self-limited diarrhea, typically attributed to the magnesium supplement and resolving within 1-2 days of discontinuation. Most adverse events were low-grade and easily managed, except for 8 serious events occurring in 5 patients. The most serious event involved a patient with recurrent Grade 4 hyponatremia despite adequate salt supplementation; this occurred in the setting of severe multifocal tumor burden with brainstem mass effect. Although hyponatremia is a known potential complication of prolonged fasting and ketogenic diets, it was uncommon in this trial and mostly mild, consistent with the MTP’s emphasis on adequate salt intake. Importantly, no serious adverse events were attributed to the MTP, suggesting that it was well-tolerated and did not add clinically significant toxicity.

Studies in glioma and GBM patients indicate that physical activity typically declines during chemoradiation and chemotherapy, largely due to treatment-related side effects such as fatigue,^41^ with little or no improvement in quality of life.^42^ In contrast, patients in this trial demonstrated a steady increase in exercise activity alongside improvements in quality of life measures. These findings align with prior evidence that metabolic therapy may improve quality of life in cancer patients, including GBM.^22^^,^^23^ The MTP may have contributed to these improvements by providing ketones, which constitute a more efficient energy source for neurons per unit of oxygen,^43^ although indirect mechanisms—such as broader metabolic effects or intentional weight loss—may also have played a role.

Survival outcomes were promising. Patients completing chemoradiation with the MTP achieved a median overall survival of 21.5 months, surpassing the 11-14 months typically reported for IDH-wildtype GBM patients receiving standard treatment,^5-7^ as well as their expected 13 months based on a recent nomogram.^44^ This is notable given that 12 of 15 patients (80%) had O‐6‐methylguanine‐DNA methyltransferase (MGMT)-unmethylated tumors, which are associated with poorer outcomes compared with MGMT-methylated cases.^2^ In comparison, control patients had a median survival of 14.7 months, aligning with both published outcomes and their nomogram-predicted survival of 14 months. Moreover, 4 of 15 patients (27%) in the MTP group achieved long-term survival (over 3 years), exceeding both the control group and historical rates (7% and 9%-11%, respectively).^45^^,^^46^ Overall, the MTP was associated with a lower risk of death compared with controls (HR = 0.42), supporting a potential survival benefit. As patients in the MTP and control groups received a comparable number of second-line treatments, these results are unlikely to be explained by subsequent interventions. They are further supported by a recent study linking therapeutically relevant GKI levels (mean 5.8) with improved outcomes in high-grade glioma patients receiving ketogenic diet therapy plus bevacizumab.^47^ Altogether, these findings suggest that the MTP may improve survival, with benefits becoming more evident beyond the first year, although this interpretation is limited by the small sample size and non-randomized design.

Despite these favorable survival outcomes, 4 of 15 patients (27%) died within 12 months, warranting further consideration. All represented clinically complex cases, including multifocal GBM, high-dose dexamethasone exposure,^32^ post-surgical ventricular entry with leptomeningeal dissemination,^48^ and Lynch syndrome, which is associated with deficient mismatch repair and temozolomide resistance.^49^ Beyond these challenges, these 4 patients also exhibited a higher mean daily GKI during chemoradiation (2.90), including the 3 highest individual GKIs observed in the trial. This may reflect lower adherence to the MTP, which in turn may be associated with shorter survival. The GKI in this subgroup was nearly twice that observed in the 11 patients with longer survival, consistent with the hypothesis that sustaining a GKI ≤2 may be relevant for optimal metabolic management in GBM.^16^

Several strengths of this trial merit emphasis. First, it enrolled a homogenous cohort of newly diagnosed IDH-wildtype GBM patients, minimizing biological heterogeneity and allowing focused evaluation of metabolic therapy in this high-risk population. Second, the trial enabled evaluation of the impact of an intensive multimodal metabolic therapy protocol that achieved high adherence and low GKI levels throughout the entire 8-9 months of standard treatment, without confounding from other off-label or lifestyle interventions. Third, outcomes were compared with a well-matched contemporary control group, treated with the same standard protocol over the same timeframe. Collectively, these features enhance internal validity and support the attribution of observed effects to the MTP.

Limitations include the small sample size, which necessitates cautious interpretation of the observed effects of the MTP on GBM outcomes. The trial’s intensive fasting and dietary requirements also limited enrollment, as some eligible patients declined the MTP—or were not offered it—due to perceived difficulty or restrictiveness, highlighting the need for refinements to improve broader applicability. Moreover, although baseline characteristics were similar, the contemporary control group was non-randomized, introducing potential self-selection bias; randomizing newly diagnosed GBM patients would have been challenging, as many cancer patients may be unwilling to risk assignment to standard treatment alone, self-selecting into the intervention arm.^50^ Furthermore, the absence of an MTP-only arm (without chemoradiation) is another limitation, which may be relevant given that both radiation and temozolomide can alter the tumor microenvironment and promote more aggressive recurrent gliomas in some contexts.^51^^,^^52^ Since standard chemoradiation remains the established backbone of care, such a design was not feasible in the current trial; however, future studies could evaluate delayed chemoradiation in selected patients when combined with intensive metabolic therapy. Finally, while the MTP incorporated prolonged fasting to optimally decrease blood glucose and glutamine levels, fasting and dietary interventions alone can only partially suppress fermentable fuels,^33^^,^^34^ and additional pharmacological strategies may be necessary to achieve greater metabolic control.^16^

In conclusion, the MTP was feasible, well-tolerated, and associated with improved exercise activity, quality of life, and survival outcomes in IDH-wildtype GBM patients, with a median overall survival of 21.5 months versus 14.7 months in contemporary controls, and a 3-year survival of 27% versus 7%. These findings suggest that integrating standard treatment with intensive multimodal ketogenic metabolic therapy may improve outcomes in GBM, though larger controlled trials are needed to confirm this.

## Supplementary Material

vdag165_Supplementary_Data

## Data Availability

The data supporting the findings of this trial are available from the corresponding author upon reasonable request.
