# Supplementary material for: Intensive multimodal ketogenic metabolic therapy in glioblastoma: A clinical trial
Source: Neurooncol Adv. 2026 Jun 29;8(1):vdag165. doi: 10.1093/noajnl/vdag165 (PMC13427763; doi:10.1093/noajnl/vdag165)
Supplement: vdag165_Supplementary_Data [file vdag165_supplementary_data.pdf]

Metabolic Therapy Program (MTP)

Waikato Glioblastoma Study

Matthew CL Phillips, Neurologist, 2021

Table Of Contents

Introduction To The Program….3

How To Do The Multiday Fasts (Must-Read Before Starting)….4

How To Do The Time-Restricted Ketogenic Diet (Must-Read Before Starting)….5

If You Eat Out….6

Your Chemoradiation Data….7

Your 4-Week Break Data….9

Your Adjuvant Chemotherapy Data….10

MTP Recipes….16

Meal Planners And Shopping Lists….91

Introduction To The Program

This booklet has all the information you need to conduct our **Metabolic Therapy Program (MTP)** for glioblastoma, a very aggressive brain cancer. Please understand, this program may be challenging, especially in the first month. The reason is that it relies on **multiday fasts** combined with a **time-restricted ketogenic diet**, which will constitute a radical lifestyle change and 100% dedication from you. However, I am confident you can do it.

Basically, the **metabolic theory of cancer** suggests that cancer may be partly driven by a **carbohydrate-rich western diet** (50% or more carbohydrates) combined with a **frequent eating pattern** (three times a day, plus snacks). Thus, over the last 5 years, we have created this MTP which aims to:

(1) Introduce **multiday (5-day) fasts** that eradicate the intake of carbohydrates (the main cancer fuel) and protein (the backup cancer fuel).

(2) Introduce a **time-restricted (one-to-two meals a day) ketogenic diet** that reduces excessive eating during the day, especially snacking (limiting the steady fuel supply to cancer cells), and limits the intake of carbohydrates (the main cancer fuel).

In addition to lowering glucose, fasting and ketogenic diets elevate **ketones**, which are energy molecules derived from fat that normal cells can use, but cancer cells probably cannot. Fasting and ketogenic diets also improve the function of **mitochondria**, the “batteries” inside cells that make energy for cells. Moreover, fasting and ketogenic diets put normal cells into a **survival mode** that increases their resilience to the toxicity of chemotherapy and radiotherapy, whereas cancer cells cannot enter this survival mode.

Understand that **it is 100% essential to treat the MTP as a therapy**. This mindset is crucial to avoiding the many temptations that may attempt to derail you as you try to improve your health.

Lastly, remember that **you are a pioneer**, participating in a unique, combined treatment approach to cancer therapy. There is a whole team of specialists at Waikato Hospital ready to support you.

If you have any **questions, concerns, or problems** on the MTP, please do not hesitate to email me at matthewclphillips@metabolicneurologist.com at any time.

Matt and the team.

How To Do The Multiday Fasts (Must-Read Before Starting)

There are **five guidelines** that will confer success with the 5-day fasts (the first two are the most critical). Please stick to all five guidelines. This will ensure that your fasts are optimized.

(1) It's now about whether you can do it, it's about whether you will do it…your body will know how to do everything.
- The hardest part of these fasts is often before they even begin. The mind starts creating all sorts of doubts beforehand, but remember that all humans evolved a natural ability to fast for days at a time (if we hadn't, we wouldn't be here). You have the power here - you can do it.

(2) Drink 2 L of the salt elixir or bone broth daily.
- In the first days of the fast, urine output increases, which makes a person more susceptible to dehydration and salt loss, which can result in a headache, constipation, nausea, cramps, and dizziness. To prevent this, drink a minimum of 2 L of salt elixir or bone broth daily, with the first 1 L in the initial 1-2 hours of the morning, and the second 1 L before 6 pm. Avoid drinking excess water after 6 pm.

(3) Take no supplements, except two magnesium tablets daily.
- You need to take the two magnesium tablets daily, which will prevent muscle aches, cramps, and improve sleep (do not take magnesium during the ketogenic diet). Please do not take any other supplements; if you deem one of them vital, please run it by me.

(4) Drink the right extra fluids (water, tea, or coffee).
- You may drink extra water, tea, or coffee on a fasting day, but they do not count as part of the minimum 2 L requirement stated above. Water may be still or sparkling, and you can add some lemon or lime juice. Tea is best on its own. Coffee is best black, but you may add 1 tsp of unsweetened almond milk, pure cream, coconut oil, or a pinch of cinnamon or nutmeg (limit coffee to 2 cups per day). And of course, do not add sugar, milk, or artificial flavours or sweeteners to any drink.

(5) Record all of your data in the program.
- Check your blood glucose and ketones every night at bedtime, then write the date as well as glucose and ketone numbers in this program. Record your weight at the start and end of each fast, as indicated in the program. We will try to avoid dexamethasone (steroid) if possible, but if you have to take it please write the dose to the left of the respective day (in mg).

Regarding **exercise**, you can do light exercise (such as a 30-minute walk) daily on the first fast. For all other fasts you may exercise more vigorously.

How To Do The Time-Restricted Ketogenic Diet (Must-Read Before Starting)

As with the fasts, there are also **five guidelines** that will confer success with the ketogenic diet (the first two are the most critical). Please understand, there is no "half-way" with the ketogenic diet. It's all or none. We have already designed the diet to be as flexible and palatable as possible, but still effective.

(1) Follow the recipes as written and eat only within your chosen meal hours per day (eat to 80% full, skip dessert or limit to 1 serve daily, no snacks between meal hours).
- It may be tempting to alter the recipes. However, I have learned through experience this won't work and virtually always lowers the ketone levels (which is disheartening for you), so do not alter the recipes in the program. If you crave variety, try multiple recipes.
- During the meal hours, you may eat any ketogenic diet recipes until you are 80% full. Please eat slowly and do not stuff yourself. You can do any combination (breakfast and lunch, lunch and dinner, etc) and the timing of the meal hours may vary from day to day, but stick to number of meals shown in the plan.
- Please limit dessert to 1-2 serves per week as sweet-tasting food, even if they lack carbohydrates, can lower ketone levels.
- Lastly, I cannot emphasize enough how critical it is to stop all snacking between the meal hours. Just stick to water, tea, and coffee.

(2) Drink at least 1.5 L of water daily, and follow the minimum salt serves in the recipes.
- Urine output increases on a ketogenic diet, resulting in dehydration and salt loss, which may lead to headache, constipation, nausea, cramps, and dizziness. So drink at least 1.5 L of extra water every day on the ketogenic diet, and follow the salt serves given in the recipes (by the way, the salt serves in the recipes are minimums - add as much salt as you like).

(3) Take no supplements, except one multivitamin daily.
- To ensure you are getting a bare minimum of vitamin levels, take one of the prescribed multivitamins daily (but do not take multivitamins during the fasts). Please stop all other supplements, including any other multivitamins.

(4) Drink the right extra fluids (water, tea, coffee, or limited red wine).
- You may drink extra water, tea, coffee, or red wine, but none of these count as part of the minimum 1.5 L water requirement stated above. Water may be still or sparkling, and you can add some lemon or lime juice. Tea is best on its own. Coffee is best black, but you may add 1 tsp of unsweetened almond milk, pure cream, coconut oil, or a pinch of cinnamon or nutmeg (limit coffee to 2 cups per day). A bit of red wine is ok, but limit red wine to half a cup (125 ml) per day), and no beer or white wine at any time.

(5) Record all of your data in the program.
- Check your blood glucose and ketones every night at bedtime, then write the date as well as glucose and ketone numbers in this program. Record your weight as indicated in the program. If you have to take dexamethasone please write the dose to the left of the respective day (in mg).

Regarding **exercise**, you can exercise as much as you like on the ketogenic diet.

If You Eat Out

Sometimes, it is good to eat out, perhaps at a restaurant or social gathering. This is ok as long as you stick to "allowable" foods. Stick to the following list of meals when eating out, and always add a side of pure fat (Hollandaise, butter, cream, aioli, sour cream, olive oil, or mayonnaise) to every meal. Even if your meal comes with a side of pure fat (for example, steak and butter), you may need to order more of the fat side - in general, you need about 3-4 tbsp of pure fat in every meal. However, the best way to optimize this is to bring your own bottle of extra virgin olive oil when you eat out, as the olive oil in most restaurants is usually tainted with refined seed oils.

Breakfast

Breakfast is relatively easy, but you must **avoid bread, hash browns, sundried tomatoes, potatoes, fruits, muesli, porridge, cereals, milk, waffles, and pancakes**. Example breakfast options include:

Eggs any style (add 3-4 tbsp Hollandaise)
Salmon and bacon (add 3-4 tbsp Hollandaise)
Avocados, olives, mushrooms (add 3-4 tbsp cream)
Halloumi cheese (add 3-4 tbsp aioli)
Omelettes containing just salmon, bacon, cheese, avocado, or spinach (add 3-4 tbsp sour cream)
Green vegetables and salads (add 3-4 tbsp olive oil)

Lunch or Dinner

Both lunch and dinner are best done by sticking to salads, **avoid breads, croutons, potatoes, pastas, sodas, wedges, chips, rice, and nachos**. Example lunch and dinner options include:

Chicken, beef, lamb, or seafood salad (add 3-4 tbsp olive oil)
Steak (add 3-4 tbsp butter, aioli, sour cream, bearnaise, or blue cheese sauce)
Fish with green vegetables (add 3-4 tbsp butter, aioli, or olive oil)
Salmon or tuna sashimi (add 3-4 tbsp yuzo mayo)
Green vegetables and leafy green salads (add 3-4 tbsp olive oil or mayo)

Dessert

Desserts at nearly any restaurant invariably contain sugar. Stick to cheese plates (just the cheese, don't eat anything else on the plate), and **avoid any of the desserts**. Truthfully, the best option is to skip dessert.

Your Chemoradiation Data

If you take <u>dexamethasone</u> (steroid), please mark the dose beside the particular day, every day.

Write the actual date you start chemoradiation (Day 1) here (D/M/Y): [ ]

Week Before Concurrent Chemoradiation - Starting 1st Fast This Week

4 Days To Go!	Glucose		Ketones		Last supper tonight	
3 Days To Go!	Glucose		Ketones		1st Fast, 1st Day	Weight
2 Days To Go!	Glucose		Ketones		1st Fast, 2nd Day	
1 Day To Go!	Glucose		Ketones		1st Fast, 3rd Day	

Week 1, Concurrent Chemoradiation - Continue 1st Fast This Week

Day 1	Glucose		Ketones		1st Fast, 4th Day	
Day 2	Glucose		Ketones		1st Fast, 5th Day	Weight
Day 3	Glucose		Ketones		Meal hour: 1st ________ 2nd ________	
Day 4	Glucose		Ketones		Meal hour: 1st ________ 2nd ________	
Day 5	Glucose		Ketones		Meal hour (one meal only): ________	
Day 6	Glucose		Ketones		Meal hour: 1st ________ 2nd ________	
Day 7	Glucose		Ketones		Meal hour: 1st ________ 2nd ________	

Week 2, Concurrent Chemoradiation

Day 8	Glucose		Ketones		Meal hour: ________
Day 9	Glucose		Ketones		Meal hour: 1st ________ 2nd ________
Day 10	Glucose		Ketones		Meal hour: ________
Day 11	Glucose		Ketones		Meal hour: 1st ________ 2nd ________
Day 12	Glucose		Ketones		Meal hour: ________
Day 13	Glucose		Ketones		Meal hour: 1st ________ 2nd ________
Day 14	Glucose		Ketones		Meal hour: 1st ________ 2nd ________

Week 3, Concurrent Chemoradiation - Starting 2nd Fast This Week

Day 15	Glucose		Ketones		Meal hour: ________	
Day 16	Glucose		Ketones		Meal hour: 1st ________ 2nd ________	
Day 17	Glucose		Ketones		Meal hour: ________	
Day 18	Glucose		Ketones		Meal hour: 1st ________ 2nd ________	
Day 19	Glucose		Ketones		2nd Fast, 1st Day	Weight
Day 20	Glucose		Ketones		2nd Fast, 2nd Day	
Day 21	Glucose		Ketones		2nd Fast, 3rd Day	

If you take <u>dexamethasone</u> (steroid), please mark the dose beside the particular day, every day.

<u>Week 4, Concurrent Chemoradiation</u> - Continue 2nd Fast This Week

Day 22	Glucose		Ketones		2nd Fast, 4th Day
Day 23	Glucose		Ketones		2nd Fast, 5th Day Weight
Day 24	Glucose		Ketones		Meal hour: 1st ________ 2nd ________
Day 25	Glucose		Ketones		Meal hour: 1st ________ 2nd ________
Day 26	Glucose		Ketones		Meal hour: ________
Day 27	Glucose		Ketones		Meal hour: 1st ________ 2nd ________
Day 28	Glucose		Ketones		Meal hour: 1st ________ 2nd ________

<u>Week 5, Concurrent Chemoradiation</u>

Day 29	Glucose		Ketones		Meal hour: ________
Day 30	Glucose		Ketones		Meal hour: 1st ________ 2nd ________
Day 31	Glucose		Ketones		Meal hour: ________
Day 32	Glucose		Ketones		Meal hour: 1st ________ 2nd ________
Day 33	Glucose		Ketones		Meal hour: ________
Day 34	Glucose		Ketones		Meal hour: 1st ________ 2nd ________
Day 35	Glucose		Ketones		Meal hour: 1st ________ 2nd ________

<u>Week 6, Concurrent Chemoradiation</u>

Day 36	Glucose		Ketones		Meal hour: ________
Day 37	Glucose		Ketones		Meal hour: 1st ________ 2nd ________
Day 38	Glucose		Ketones		Meal hour: ________
Day 39	Glucose		Ketones		Meal hour: 1st ________ 2nd ________
Day 40	Glucose		Ketones		Meal hour: ________
Day 41	Glucose		Ketones		Meal hour: 1st ________ 2nd ________
Day 42	Glucose		Ketones		Meal hour: 1st ________ 2nd ________

Write the actual date of Day 42 here (D/M/Y): ☐ **Day 42 Weight** ☐

Your 4-Week Break Data

If you take <u>dexamethasone</u> (steroid), please mark the dose beside the particular day, every day.

<u>**Week 1, 4-Week Break**</u>

Day 1	Glucose		Ketones		Meal hour: ________
Day 2	Glucose		Ketones		Meal hour: 1st ________ 2nd ________
Day 3	Glucose		Ketones		Meal hour: ________
Day 4	Glucose		Ketones		Meal hour: 1st ________ 2nd ________
Day 5	Glucose		Ketones		Meal hour: ________
Day 6	Glucose		Ketones		Meal hour: 1st ________ 2nd ________
Day 7	Glucose		Ketones		Meal hour: 1st ________ 2nd ________

<u>**Week 2, 4-Week Break**</u>

Day 8	Glucose		Ketones		Meal hour: ________
Day 9	Glucose		Ketones		Meal hour: 1st ________ 2nd ________
Day 10	Glucose		Ketones		Meal hour: ________
Day 11	Glucose		Ketones		Meal hour: 1st ________ 2nd ________
Day 12	Glucose		Ketones		Meal hour: ________
Day 13	Glucose		Ketones		Meal hour: 1st ________ 2nd ________
Day 14	Glucose		Ketones		Meal hour: 1st ________ 2nd ________

<u>**Week 3, 4-Week Break**</u>

Day 15	Glucose		Ketones		Meal hour: ________
Day 16	Glucose		Ketones		Meal hour: 1st ________ 2nd ________
Day 17	Glucose		Ketones		Meal hour: ________
Day 18	Glucose		Ketones		Meal hour: 1st ________ 2nd ________
Day 19	Glucose		Ketones		Meal hour: ________
Day 20	Glucose		Ketones		Meal hour: 1st ________ 2nd ________
Day 21	Glucose		Ketones		Meal hour: 1st ________ 2nd ________

<u>**Week 4, 4-Week Break**</u> - **Starting Cycle 1 Fast This Week**

Day 22	Glucose		Ketones		Meal hour: ________
Day 23	Glucose		Ketones		Meal hour: 1st ________ 2nd ________
Day 24	Glucose		Ketones		Meal hour: ________
Day 25	Glucose		Ketones		Meal hour: 1st ________ 2nd ________
Day 26	Glucose		Ketones		Cycle 1 Fast, 1st Day Weight
Day 27	Glucose		Ketones		Cycle 1 Fast, 2nd Day
Day 28	Glucose		Ketones		Cycle 1 Fast, 3rd Day

Your Adjuvant Chemotherapy Data

If you take dexamethasone (steroid), please mark the dose beside the particular day, every day.

Week 1, Cycle 1 of Adjuvant Chemotherapy - Chemo Week (C) + Continue Cycle 1 Fast

Day 1 (C)	Glucose		Ketones		Cycle 1 Fast, 4th Day	
Day 2 (C)	Glucose		Ketones		Cycle 1 Fast, 5th Day	Weight
Day 3 (C)	Glucose		Ketones		Meal hour: 1st _________ 2nd _________	
Day 4 (C)	Glucose		Ketones		Meal hour: 1st _________ 2nd _________	
Day 5 (C)	Glucose		Ketones		Meal hour: _________	
Day 6	Glucose		Ketones		Meal hour: 1st _________ 2nd _________	
Day 7	Glucose		Ketones		Meal hour: 1st _________ 2nd _________	

Week 2, Cycle 1 of Adjuvant Chemotherapy

Day 8	Glucose		Ketones		Meal hour: _________
Day 9	Glucose		Ketones		Meal hour: 1st _________ 2nd _________
Day 10	Glucose		Ketones		Meal hour: _________
Day 11	Glucose		Ketones		Meal hour: 1st _________ 2nd _________
Day 12	Glucose		Ketones		Meal hour: _________
Day 13	Glucose		Ketones		Meal hour: 1st _________ 2nd _________
Day 14	Glucose		Ketones		Meal hour: 1st _________ 2nd _________

Week 3, Cycle 1 of Adjuvant Chemotherapy

Day 15	Glucose		Ketones		Meal hour: _________
Day 16	Glucose		Ketones		Meal hour: 1st _________ 2nd _________
Day 17	Glucose		Ketones		Meal hour: _________
Day 18	Glucose		Ketones		Meal hour: 1st _________ 2nd _________
Day 19	Glucose		Ketones		Meal hour: _________
Day 20	Glucose		Ketones		Meal hour: 1st _________ 2nd _________
Day 21	Glucose		Ketones		Meal hour: 1st _________ 2nd _________

Week 4, Cycle 1 of Adjuvant Chemotherapy - Starting Cycle 2 Fast This Week

Day 22	Glucose		Ketones		Meal hour: _________	
Day 23	Glucose		Ketones		Meal hour: 1st _________ 2nd _________	
Day 24	Glucose		Ketones		Meal hour: _________	
Day 25	Glucose		Ketones		Meal hour: 1st _________ 2nd _________	
Day 26	Glucose		Ketones		Cycle 2 Fast, 1st Day	Weight
Day 27	Glucose		Ketones		Cycle 2 Fast, 2nd Day	
Day 28	Glucose		Ketones		Cycle 2 Fast, 3rd Day	

If you take *dexamethasone* (steroid), please mark the dose beside the particular day, every day.

Week 1, Cycle 2 of Adjuvant Chemotherapy - Chemo Week (C) + Continue Cycle 2 Fast

Day 1 (C)	Glucose		Ketones		Cycle 2 Fast, 4th Day	
Day 2 (C)	Glucose		Ketones		Cycle 2 Fast, 5th Day	Weight
Day 3 (C)	Glucose		Ketones		Meal hour: 1st __________ 2nd __________	
Day 4 (C)	Glucose		Ketones		Meal hour: 1st __________ 2nd __________	
Day 5 (C)	Glucose		Ketones		Meal hour: __________	
Day 6	Glucose		Ketones		Meal hour: 1st __________ 2nd __________	
Day 7	Glucose		Ketones		Meal hour: 1st __________ 2nd __________	

Week 2, Cycle 2 of Adjuvant Chemotherapy

Day 8	Glucose		Ketones		Meal hour: __________
Day 9	Glucose		Ketones		Meal hour: 1st __________ 2nd __________
Day 10	Glucose		Ketones		Meal hour: __________
Day 11	Glucose		Ketones		Meal hour: 1st __________ 2nd __________
Day 12	Glucose		Ketones		Meal hour: __________
Day 13	Glucose		Ketones		Meal hour: 1st __________ 2nd __________
Day 14	Glucose		Ketones		Meal hour: 1st __________ 2nd __________

Week 3, Cycle 2 of Adjuvant Chemotherapy

Day 15	Glucose		Ketones		Meal hour: __________
Day 16	Glucose		Ketones		Meal hour: 1st __________ 2nd __________
Day 17	Glucose		Ketones		Meal hour: __________
Day 18	Glucose		Ketones		Meal hour: 1st __________ 2nd __________
Day 19	Glucose		Ketones		Meal hour: __________
Day 20	Glucose		Ketones		Meal hour: 1st __________ 2nd __________
Day 21	Glucose		Ketones		Meal hour: 1st __________ 2nd __________

Week 4, Cycle 2 of Adjuvant Chemotherapy - Starting Cycle 3 Fast This Week

Day 22	Glucose		Ketones		Meal hour: __________	
Day 23	Glucose		Ketones		Meal hour: 1st __________ 2nd __________	
Day 24	Glucose		Ketones		Meal hour: __________	
Day 25	Glucose		Ketones		Meal hour: 1st __________ 2nd __________	
Day 26	Glucose		Ketones		Cycle 3 Fast, 1st Day	Weight
Day 27	Glucose		Ketones		Cycle 3 Fast, 2nd Day	
Day 28	Glucose		Ketones		Cycle 3 Fast, 3rd Day	

If you take <u>dexamethasone</u> (steroid), please mark the dose beside the particular day, every day.

<u>Week 1, Cycle 3 of Adjuvant Chemotherapy</u>- Chemo Week (C) + Continue Cycle 3 Fast

Day 1 (C)	Glucose		Ketones		Cycle 3 Fast, 4th Day
Day 2 (C)	Glucose		Ketones		Cycle 3 Fast, 5th Day Weight ____
Day 3 (C)	Glucose		Ketones		Meal hour: 1st ________ 2nd ________
Day 4 (C)	Glucose		Ketones		Meal hour: 1st ________ 2nd ________
Day 5 (C)	Glucose		Ketones		Meal hour: ________
Day 6	Glucose		Ketones		Meal hour: 1st ________ 2nd ________
Day 7	Glucose		Ketones		Meal hour: 1st ________ 2nd ________

<u>Week 2, Cycle 3 of Adjuvant Chemotherapy</u>

Day 8	Glucose		Ketones		Meal hour: ________
Day 9	Glucose		Ketones		Meal hour: 1st ________ 2nd ________
Day 10	Glucose		Ketones		Meal hour: ________
Day 11	Glucose		Ketones		Meal hour: 1st ________ 2nd ________
Day 12	Glucose		Ketones		Meal hour: ________
Day 13	Glucose		Ketones		Meal hour: 1st ________ 2nd ________
Day 14	Glucose		Ketones		Meal hour: 1st ________ 2nd ________

<u>Week 3, Cycle 3 of Adjuvant Chemotherapy</u>

Day 15	Glucose		Ketones		Meal hour: ________
Day 16	Glucose		Ketones		Meal hour: 1st ________ 2nd ________
Day 17	Glucose		Ketones		Meal hour: ________
Day 18	Glucose		Ketones		Meal hour: 1st ________ 2nd ________
Day 19	Glucose		Ketones		Meal hour: ________
Day 20	Glucose		Ketones		Meal hour: 1st ________ 2nd ________
Day 21	Glucose		Ketones		Meal hour: 1st ________ 2nd ________

<u>Week 4, Cycle 3 of Adjuvant Chemotherapy</u> - Starting Cycle 4 Fast This Week

Day 22	Glucose		Ketones		Meal hour: ________
Day 23	Glucose		Ketones		Meal hour: 1st ________ 2nd ________
Day 24	Glucose		Ketones		Meal hour: ________
Day 25	Glucose		Ketones		Meal hour: 1st ________ 2nd ________
Day 26	Glucose		Ketones		Cycle 4 Fast, 1st Day Weight ____
Day 27	Glucose		Ketones		Cycle 4 Fast, 2nd Day
Day 28	Glucose		Ketones		Cycle 4 Fast, 3rd Day

If you take <u>dexamethasone</u> (steroid), please mark the dose beside the particular day, every day.

<u>Week 1, Cycle 4 of Adjuvant Chemotherapy</u> - Chemo Week (C) + Continue Cycle 4 Fast

Day 1 (C)	Glucose		Ketones		Cycle 4 Fast, 4th Day		
Day 2 (C)	Glucose		Ketones		Cycle 4 Fast, 5th Day	Weight	
Day 3 (C)	Glucose		Ketones		Meal hour: 1st __________	2nd __________	
Day 4 (C)	Glucose		Ketones		Meal hour: 1st __________	2nd __________	
Day 5 (C)	Glucose		Ketones		Meal hour: __________		
Day 6	Glucose		Ketones		Meal hour: 1st __________	2nd __________	
Day 7	Glucose		Ketones		Meal hour: 1st __________	2nd __________	

<u>Week 2, Cycle 4 of Adjuvant Chemotherapy</u>

Day 8	Glucose		Ketones		Meal hour: __________	
Day 9	Glucose		Ketones		Meal hour: 1st __________	2nd __________
Day 10	Glucose		Ketones		Meal hour: __________	
Day 11	Glucose		Ketones		Meal hour: 1st __________	2nd __________
Day 12	Glucose		Ketones		Meal hour: __________	
Day 13	Glucose		Ketones		Meal hour: 1st __________	2nd __________
Day 14	Glucose		Ketones		Meal hour: 1st __________	2nd __________

<u>Week 3, Cycle 4 of Adjuvant Chemotherapy</u>

Day 15	Glucose		Ketones		Meal hour: __________	
Day 16	Glucose		Ketones		Meal hour: 1st __________	2nd __________
Day 17	Glucose		Ketones		Meal hour: __________	
Day 18	Glucose		Ketones		Meal hour: 1st __________	2nd __________
Day 19	Glucose		Ketones		Meal hour: __________	
Day 20	Glucose		Ketones		Meal hour: 1st __________	2nd __________
Day 21	Glucose		Ketones		Meal hour: 1st __________	2nd __________

<u>Week 4, Cycle 4 of Adjuvant Chemotherapy</u> - Starting Cycle 5 Fast This Week

Day 22	Glucose		Ketones		Meal hour: __________		
Day 23	Glucose		Ketones		Meal hour: 1st __________	2nd __________	
Day 24	Glucose		Ketones		Meal hour: __________		
Day 25	Glucose		Ketones		Meal hour: 1st __________	2nd __________	
Day 26	Glucose		Ketones		Cycle 5 Fast, 1st Day	Weight	
Day 27	Glucose		Ketones		Cycle 5 Fast, 2nd Day		
Day 28	Glucose		Ketones		Cycle 5 Fast, 3rd Day		

If you take <u>dexamethasone</u> (steroid), please mark the dose beside the particular day, every day.

<u>Week 1, Cycle 5 of Adjuvant Chemotherapy</u> - Chemo Week (C) + Continue Cycle 5 Fast

Day 1 (C)	Glucose		Ketones		Cycle 5 Fast, 4th Day	
Day 2 (C)	Glucose		Ketones		Cycle 5 Fast, 5th Day	Weight
Day 3 (C)	Glucose		Ketones		Meal hour: 1st __________ 2nd __________	
Day 4 (C)	Glucose		Ketones		Meal hour: 1st __________ 2nd __________	
Day 5 (C)	Glucose		Ketones		Meal hour: __________	
Day 6	Glucose		Ketones		Meal hour: 1st __________ 2nd __________	
Day 7	Glucose		Ketones		Meal hour: 1st __________ 2nd __________	

<u>Week 2, Cycle 5 of Adjuvant Chemotherapy</u>

Day 8	Glucose		Ketones		Meal hour: __________
Day 9	Glucose		Ketones		Meal hour: 1st __________ 2nd __________
Day 10	Glucose		Ketones		Meal hour: __________
Day 11	Glucose		Ketones		Meal hour: 1st __________ 2nd __________
Day 12	Glucose		Ketones		Meal hour: __________
Day 13	Glucose		Ketones		Meal hour: 1st __________ 2nd __________
Day 14	Glucose		Ketones		Meal hour: 1st __________ 2nd __________

<u>Week 3, Cycle 5 of Adjuvant Chemotherapy</u>

Day 15	Glucose		Ketones		Meal hour: __________
Day 16	Glucose		Ketones		Meal hour: 1st __________ 2nd __________
Day 17	Glucose		Ketones		Meal hour: __________
Day 18	Glucose		Ketones		Meal hour: 1st __________ 2nd __________
Day 19	Glucose		Ketones		Meal hour: __________
Day 20	Glucose		Ketones		Meal hour: 1st __________ 2nd __________
Day 21	Glucose		Ketones		Meal hour: 1st __________ 2nd __________

<u>Week 4, Cycle 5 of Adjuvant Chemotherapy</u> - Starting Cycle 6 Fast This Week

Day 22	Glucose		Ketones		Meal hour: __________	
Day 23	Glucose		Ketones		Meal hour: 1st __________ 2nd __________	
Day 24	Glucose		Ketones		Meal hour: __________	
Day 25	Glucose		Ketones		Meal hour: 1st __________ 2nd __________	
Day 26	Glucose		Ketones		Cycle 6 Fast, 1st Day	Weight
Day 27	Glucose		Ketones		Cycle 6 Fast, 2nd Day	
Day 28	Glucose		Ketones		Cycle 6 Fast, 3rd Day	

If you take <u>dexamethasone</u> (steroid), please mark the dose beside the particular day, every day.

<u>Week 1, Cycle 6 of Adjuvant Chemotherapy</u> - Chemo Week (C) + Continue Cycle 6 Fast

Day 1 (C)	Glucose		Ketones		Cycle 6 Fast, 4th Day
Day 2 (C)	Glucose		Ketones		Cycle 6 Fast, 5th Day Weight
Day 3 (C)	Glucose		Ketones		Meal hour: 1st __________ 2nd __________
Day 4 (C)	Glucose		Ketones		Meal hour: 1st __________ 2nd __________
Day 5 (C)	Glucose		Ketones		Meal hour: __________
Day 6	Glucose		Ketones		Meal hour: 1st __________ 2nd __________
Day 7	Glucose		Ketones		Meal hour: 1st __________ 2nd __________

<u>Week 2, Cycle 6 of Adjuvant Chemotherapy</u>

Day 8	Glucose		Ketones		Meal hour: __________
Day 9	Glucose		Ketones		Meal hour: 1st __________ 2nd __________
Day 10	Glucose		Ketones		Meal hour: __________
Day 11	Glucose		Ketones		Meal hour: 1st __________ 2nd __________
Day 12	Glucose		Ketones		Meal hour: __________
Day 13	Glucose		Ketones		Meal hour: 1st __________ 2nd __________
Day 14	Glucose		Ketones		Meal hour: 1st __________ 2nd __________

<u>Week 3, Cycle 6 of Adjuvant Chemotherapy</u>

Day 15	Glucose		Ketones		Meal hour: __________
Day 16	Glucose		Ketones		Meal hour: 1st __________ 2nd __________
Day 17	Glucose		Ketones		Meal hour: __________
Day 18	Glucose		Ketones		Meal hour: 1st __________ 2nd __________
Day 19	Glucose		Ketones		Meal hour: __________
Day 20	Glucose		Ketones		Meal hour: 1st __________ 2nd __________
Day 21	Glucose		Ketones		Meal hour: 1st __________ 2nd __________

<u>Week 4, Cycle 6 of Adjuvant Chemotherapy</u> - Starting Cycle 7 Fast This Week (If More Chemo Planned)

Day 22	Glucose		Ketones		Meal hour: __________
Day 23	Glucose		Ketones		Meal hour: 1st __________ 2nd __________
Day 24	Glucose		Ketones		Meal hour: __________
Day 25	Glucose		Ketones		Meal hour: 1st __________ 2nd __________
Day 26	Glucose		Ketones		Cycle 7 Fast, 1st Day Weight
Day 27	Glucose		Ketones		Cycle 7 Fast, 2nd Day
Day 28	Glucose		Ketones		Cycle 7 Fast, 3rd Day

MTP Recipes

THE FASTS..17
Salt Elixir..18
Bone Broth..19

EARLY RISERS..20
Avocado Smoothie..21
Berry Smoothie..22
Scrambled Eggs..23
Eggs Hollandaise..24
Keto Porridge..25
Goat Cheese Omelette..26
Kale, Eggs, & Chorizo..27
Keto Granola..28
Philly Cheese Steak Omelette..29
Shakshuka..30
Bacon Avocado Muffins..31
Chia Seed Parfaits..32
Vanilla Pecan Bars..33

SALADS..34
Caprese Salad..35
Strawberry Salad..36
Mediterranean Salad..37
Greek Salad..38
Broccoli & Bacon Salad..39
Smoked Salmon Salad..40
Basil Pesto Chicken Salad..41

STEWS & SOUPS..42
Garden Chicken..43
Rustic Beef Stew..44
Hamburger Soup..45
Thai Chicken Soup..46

BURGERS & BREADS..47
Feta Burgers..48
Cheeseburgers..49
Chicken Sandwiches..50
Keto Tortillas..51
Keto Crackers..52
Keto Bread..53
Keto Nutloaf..54

EUROPEAN..55
Slow-Cooked Lamb..56
Tuscan Salmon..57
Caprese Chicken..58
Lemony Gurnard..59
Keto Pizza..60
Salmon Fry..61
Greek Chicken..62

PAN-AMERICAN..63
Salsa Salmon..64
Bacon Avocado Wedges..65
Worcester Steaks..66
Keto Enchiladas..67
Chicken Drumsticks..68
Steak Fajitas..69
Roast Pork..70

ASIAN..71
Palak Paneer..72
Miti Fish..73
Chicken Korma..74
Beef & Cashew Stirfry..75
Paneer Makhanwala..76
Thai Fish Curry..77
Sticky Chicken Stirfry..78
Lamb Curry..79
Indian Eggplant..80

VEGGIE SIDES..81
Marinated Olives..82
Cauliflower Rice..83
Buttered Broccoli..84
Garlic Asparagus..85
Worcester Brussels Sprouts..86
Vegetable Medley..87
Rukau..88

DESSERT..89
Fruit & Cream..90

The Fasts

Salt Elixir

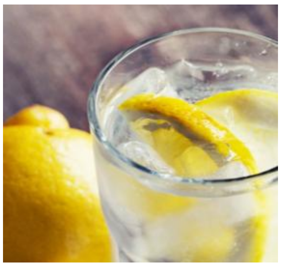

Makes 1 serve (1 serve = daily minimum electrolyte requirements for 1 day of fasting).
Preparation time: SHORT (<15 minutes).

2 L water
1 tsp salt
Juice from 1-2 lemons

(1) Add all ingredients to a blender (if the blender can only hold 1 L of liquid, you will have to do this twice during the day).
(2) Blend together and serve.

PER SERVE:

**Calories**	**Negligible**
**Fat**	**Negligible**
**Protein**	**Negligible**
**Fibre**	**Negligible**
**Net carb**	**Negligible**

Bone Broth

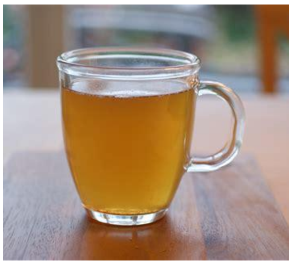

Makes 3 serves (1 serve = daily minimum electrolyte requirements for 1 day of fasting).
Preparation time: VERY LONG (>45 minutes; but nearly all cooking time).

6 L water
2 tbsp apple cider vinegar
1 kg animal bones (fish, chicken, turkey, beef, or pork)
1 brown onion
3 carrots
10 stalks celery
2 capsicum
1 tbsp (each) of salt and black pepper
Any fresh or dried herbs of your choice (thyme, parsley, and oregano are good)

(1) Add the cold water and vinegar to a large pot, followed by the bones. Let the bones sit for 30 minutes; slice the vegetables while they soak.
(2) Add sliced vegetables, salt and pepper, and any dried herbs to the water. Heat the water on medium-high heat until it nearly boils, then reduce heat to low. Simmer depending on bones: 4-8 hours (fish bones), 18-24 hours (poultry bones), or 24-48 hours (beef and pork bones).
(3) When 30 minutes are remaining, add fresh herbs. When done, remove from heat and cool for 30 minutes. Strain out all the vegetables, bones, and fat.
(4) If you lost some of the water while making the broth, top it up with extra water to return it to the original 6 L. You can store the broth in the fridge for up to 5 days.

PER SERVE:

**Calories**	**Negligible**
**Fat**	**Negligible**
**Protein**	**Negligible**
**Fibre**	**Negligible**
**Net carb**	**Negligible**

Early Risers

Avocado Smoothie

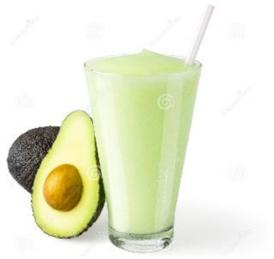

Makes 1 serve.
Preparation time: SHORT (<15 minutes).

1 avocado
1 cup canned coconut cream
1/3 cup water
1 tsp vanilla extract
1/2 tsp Natvia
1 ice cube (optional)

(1) Place all ingredients in a blender and pulse until smooth.
(2) Pour into a mug, serve, and enjoy.

PER SERVE:

**Calories**	**553**
**Fat**	**52.3 g (20.3 g saturated)**
**Protein**	**6.7 g**
**Fibre**	**17.2 g**
**Net carb**	**4.6 g**

Berry Smoothie

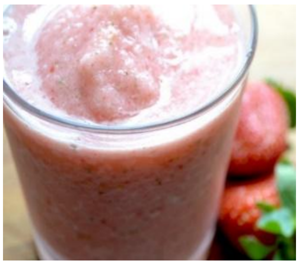

Makes 1 serve.
Preparation time: SHORT (<15 minutes).

2 strawberries OR 8 raspberries OR 8 blackberries OR 8 blueberries
1 cup canned coconut cream
1/3 cup water
1 tbsp chia seeds
1 tsp vanilla extract
1/2 tsp Natvia
1 ice cube (optional)

(1) Combine all ingredients in a blender and pulse until smooth.
(2) Pour into a mug, serve, and enjoy.

PER SERVE:

**Calories**	**308**
**Fat**	**27.2 g (16.4 g saturated)**
**Protein**	**5.0 g**
**Fibre**	**9.5 g**
**Net carb**	**3.0 g**

Scrambled Eggs

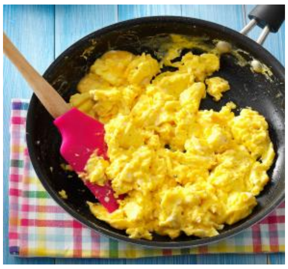

Makes 1 serve.
Preparation time: SHORT (<15 minutes).

3 eggs
1½ tbsp butter
1/2 tsp salt, pepper to taste

(1) Beat the eggs together in a bowl.
(2) Melt the butter in a pan over low heat, then add the egg mixture.
(3) Using a spatula, gently pull egg mixture to the center of the pan and let the liquid parts run out to the perimeter. Cook, continually moving eggs with the spatula, until eggs are set (this will take 2-3 minutes).
(4) Season with salt and pepper and serve.

PER SERVE:

**Calories**	**392**
**Fat**	**33.2 g (16.5 g saturated)**
**Protein**	**21.2 g**
**Fibre**	**0 g**
**Net carb**	**1.2 g**

Eggs Hollandaise

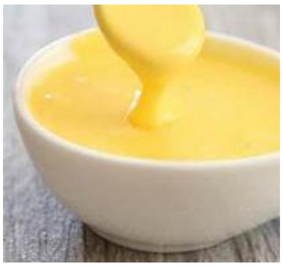

Makes 2 serves.
Preparation time: SHORT (<15 minutes).

2 tbsp butter
Juice from 1 lemon
2 egg yolks
1/4 cup pouring cream
1/2 tsp mustard powder
1/2 tsp salt
4 eggs

(1) Melt the butter in a double boiler (if you don't have this, use a small pot on low heat and just be careful not to burn anything from here on). Start boiling some water in a second pot as well, you will use this pot to poach the eggs.
(2) Add lemon juice, yolks, and cream to the boiler and stir constantly until thick and smooth. Do not boil or the sauce will curdle. Remove from heat and stir in the mustard powder and half the salt. Stir until smooth, then set aside while you finish the eggs.
(3) Stir in the remaining salt to the second pot when it reaches boiling, then use a spoon to stir the boiling water in a large, circular motion. When it is swirling like a tornado, add each egg one at a time to cook each egg 2-3 minutes at a time. Use a slotted spoon to remove each egg to a plate after it is cooked.
(4) Pour the Hollandaise sauce over the eggs and serve.

PER SERVE:

**Calories**	**421**
**Fat**	**37.8 g (19.4 g saturated)**
**Protein**	**17.6 g**
**Fibre**	**0 g**
**Net carb**	**2.2 g**

Keto Porridge

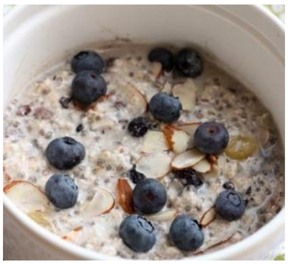

Makes 2 serves.
Preparation time: SHORT (<15 minutes).

Porridge:
1 cup canned coconut cream
3 tbsp chia seeds
3 tbsp ground flaxseed
1 tbsp pumpkin seeds
1 tsp Natvia
1/2 tsp ground cinnamon
Pinch of salt

Toppings (add the following PER SERVE):
1 strawberry OR 4 raspberries OR 4 blueberries
1/4 cup canned coconut cream (or pouring cream)

(1) Mix all porridge ingredients in a saucepan and bring to a boil on high heat, stirring constantly to prevent porridge from sticking to the bottom of the pot.
(2) Lower the heat and simmer 1-2 minutes, until desired thickness is reached.
(3) Slice up the berries if needed, add the toppings, and serve.

PER SERVE:

**Calories**	**380**
**Fat**	**31.6 g (13.7 g saturated)**
**Protein**	**9.0 g**
**Fibre**	**14.9 g**
**Net carb**	**3.5 g**

Goat Cheese Omelette

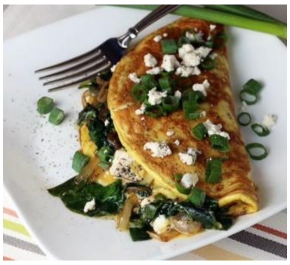

Makes 1 serve.
Preparation time: MODERATE (15-30 minutes).

2 tbsp butter
1/4 brown onion
2 stalks spring onions
60 g (1/2 bag) spinach (or kale) leaves
3 eggs
2 tbsp pouring cream
1/2 tsp salt, pepper to taste
30 g goat feta cheese

(1) Melt the butter in a pan over medium heat. Add the sliced onions to the pan and saute for 2-3 minutes.
(2) Add the spinach until it wilts; let the vegetables soak up all the butter, then transfer them to a plate. Leave the pan on the stove, but reduce heat to low-medium.
(3) Mix together the eggs, cream, and salt and pepper in a separate bowl. Pour this mixture into the pan and let the omelette cook.
(4) Once the omelette edges begin to set, spoon the vegetables over one half of the omelette, then crumble the feta cheese over the vegetables.
(5) When the top of the omelette begins to set, fold it over, then serve.

PER SERVE:

**Calories**	**654**
**Fat**	**56.7 g (33.9 g saturated)**
**Protein**	**29.7 g**
**Fibre**	**1.8 g**
**Net carb**	**5.6 g**

Kale, Eggs, & Chorizo

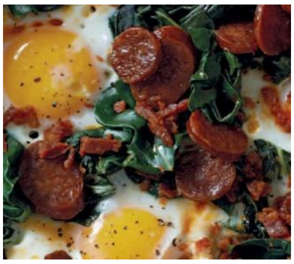

Makes 2 serves.
Preparation time: MODERATE (15-30 minutes)

3 tbsp butter
1 chorizo sausage
60 g (1/2 bag) kale leaves
3 eggs
1/2 tsp salt, pepper to taste

(1) Heat the butter in a pan over medium heat. Gently fry sliced chorizo until golden brown, then remove to a plate but leave the oil in the pan.
(2) Fry the kale in the pan until it begins to wilt. When it does, shape the kale into a nest and crack the eggs on top of it.
(3) Cook 3 minutes or until the eggs are opaque. Slide the kale nest (plus eggs) onto the plate you will eat from, then place the chorizo slices over top.
(4) Drizzle any remaining butter from the pan over top, add salt and pepper, and serve.

PER SERVE:

**Calories**	**552**
**Fat**	**48.3 g (22.3 g saturated)**
**Protein**	**26.0 g**
**Fibre**	**0.7 g**
**Net carb**	**2.1 g**

Keto Granola

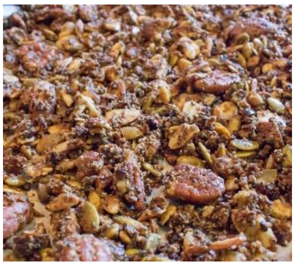

Makes 12 serves; 1 serve = (approximately) 1/2 cup granola
Preparation time: MODERATE (15-30 minutes).

Granola:
1 cup macadamia nuts
1 cup Brazil nuts
1 cup mixed nuts (any mix of walnuts, pecans, and almonds)
2 tbsp (each) of pumpkin seeds, sunflower seeds, chia seeds, and ground flaxseed
1/2 cup almond meal
1 egg
1½ cup butter (or extra virgin coconut oil), melted
1 tbsp pure vanilla extract
1 tbsp Natvia

Toppings (add the following PER SERVE):
1 strawberry OR 4 raspberries OR 4 blueberries
1/4 cup canned coconut cream (or pouring cream)

(1) Preheat oven to 180 C. Partially crush the nuts in a plastic bag with a rolling pin (optional, you can leave them whole if you prefer).
(2) Mix all the granola ingredients into a large bowl, then smooth them into a flat even layer on a baking tray greased with butter. Place in the oven for 10 minutes.
(3) Pull the granola out of the oven and mix it around, making sure the edges don't burn; place in the oven another 5-10 minutes, or until the granola has turned golden brown all over.
(4) Slice up the berries if needed, add the toppings, and serve.

PER SERVE:

**Calories**	**387**
**Fat**	**38.0 g (11.5 g saturated)**
**Protein**	**7.5 g**
**Fibre**	**5.2 g**
**Net carb**	**3.5 g**

Philly Cheese Steak Omelette

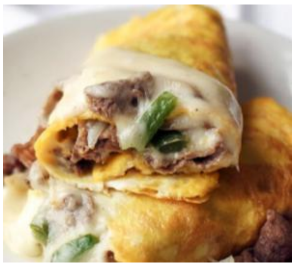

Makes 2 serves.
Preparation time: MODERATE (15-30 minutes)

1/4 cup butter
1/4 brown onion
1/2 green capsicum
200 g scotch fillet steak
1 tsp salt, pepper to taste
4 eggs
2 slices provolone (or gouda) cheese

(1) Melt half the butter in a pan over medium heat. Slice the onion, capsicum, and steak into bite-size pieces and saute in the butter for 5-6 minutes. Season with salt and pepper.
(2) While the vegetables and steak cook, melt the rest of the butter and mix it in with the eggs, salt, and pepper in a large bowl.
(3) Once the vegetables and steak are cooked, transfer them to a plate (you can cover them up to keep them warm). Leave the pan on the stove, but reduce heat to low-medium.
(4) Pour half the egg mixture into the pan and let the omelette cook. Once the edges begin to set, spoon half the vegetables and steak over one half of the omelette, followed by a slice of cheese on top. Let the cheese melt a bit. When the top of the omelette begins to set, fold it over and place on a plate.
(5) Repeat step (4) to make the second omelette, and serve.

PER SERVE:

**Calories**	**678**
**Fat**	**52.1 g (27.4 g saturated)**
**Protein**	**48.7 g**
**Fibre**	**0.8 g**
**Net carb**	**3.3 g**

Shakshuka

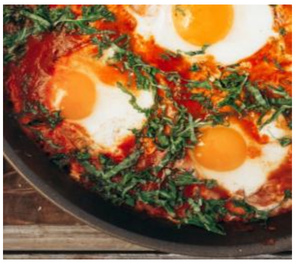

Makes 3 serves.
Preparation time: MODERATE (15-30 minutes).

3 tbsp extra virgin olive oil (or butter)
3 tbsp butter
1/2 brown onion
1 capsicum (any colour)
4 cloves garlic (or 4 tsp crushed garlic)
1 tsp salt, pepper to taste
2 tsp (each) of ground cumin and Moroccan seasoning
400 g (1 can) canned tomatoes, whole with juice
6 eggs
1/2 bunch fresh parsley (or coriander), chopped
100 g goat or cow feta cheese

(1) Heat olive oil and butter in a pan over medium (NOT high) heat. Meanwhile, chop and prepare the onion, capsicum, and garlic.
(2) Cook the onion and capsicum in the pan for 5-6 minutes, until the vegetables are soft and have soaked up as much oil and butter as possible. Then stir in the garlic for another 1-2 minutes.
(3) Season heavily with salt and pepper, then stir in the spices and tomatoes along with their juice. Simmer uncovered for 10-15 minutes, or until the sauce is thick enough to hold an indentation from the back of a spoon.
(4) Make six indentations in the sauce and crack a whole egg into each one. Cover and cook 3-5 minutes, or until the egg whites are set.
(5) Scatter parsley over top, crumble over the feta cheese, and serve.

PER SERVE:

**Calories**	**513**
**Fat**	**42.4 g (17.2 g saturated)**
**Protein**	**20.9 g**
**Fibre**	**3.1 g**
**Net carb**	**6.1 g**

Bacon Avocado Muffins

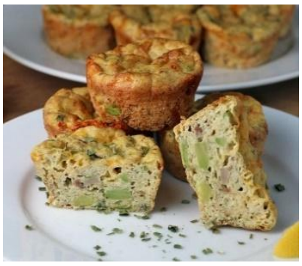

Makes 6 serves (12 muffins) total; 1 serve = 2 muffins.
Preparation time: LONG (30-45 minutes).

1/2 cup almond meal
1/4 cup ground flaxseed
1½ tbsp psyllium husk
1½ cups canned coconut cream
Juice from 1 lemon
2 tbsp butter
6 strips streaky bacon
2 avocados
30 g cheddar cheese
3 stalks spring onion
6 eggs
1 clove garlic (or 1 tsp crushed garlic)
1 tbsp coriander
1 tsp baking powder
1 tsp salt, pepper to taste

(1) Preheat the oven to 180 C. Mix together almond meal, flaxseed, psyllium husk, coconut cream, and lemon juice in a large bowl. Set aside.
(2) Add the butter to a pan and cook the sliced bacon over medium heat to your liking. Add the bacon to the mixture in the bowl.
(3) Slice/grate the avocados, cheese, and spring onions, then add to the large bowl. Following this, add the eggs, garlic, coriander, and baking powder to the mixture in the bowl to create the batter.
(4) Measure the batter between 12 greased muffin cups and bake 20-30 minutes; after 20 minutes, check every 2-3 minutes to make sure you get the baking time right. Remove when the tops of the muffins are brown, let cool, and serve.

PER SERVE:

**Calories**	**452**
**Fat**	**39.6 g (14.1 g saturated)**
**Protein**	**17.4 g**
**Fibre**	**7.0 g**
**Net carb**	**2.4 g**

Chia Seed Parfaits

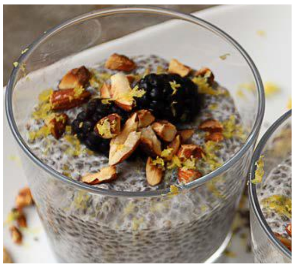

Makes 4 serves.
Preparation time: VERY LONG (>45 minutes; but nearly all fridge time).

1½ cups canned coconut cream
1 tbsp pure vanilla extract
1/2 tsp salt
1/2 cup chia seeds
1/4 cup macadamia nuts, sliced
1/4 cup Brazil nuts (or walnuts, pecans, or almonds), sliced
1 tbsp pumpkin seeds
1/2 cup berries (any combination of strawberry halves, raspberries, and blackberries)

(1) Have four jars or cups with lids ready, each about 200-250 mls in volume.
(2) Combine coconut cream with the vanilla and salt in a large bowl, then whisk in the chia seeds. Combine the nuts and pumpkin seeds in a separate small bowl.
(3) Divide half the berries into four equal parts, placing each part in the bottom of a jar. Then divide half the chia mixture into four equal parts, placing each on top of the berries in each jar. Then divide half the nut mixture into four equal parts, placing each on top of the chia mixture in each jar.
(4) Repeat step (3) with the rest of the berries, chia mixture, and nut mixture once so that each jar is full. Cover and refrigerate for at least 1-2 hours (ideally, overnight).
(5) Serve cold or at room temperature.

PER SERVE:

**Calories**	**356**
**Fat**	**30.1 g (9.4 g saturated)**
**Protein**	**7.9 g**
**Fibre**	**13.8 g**
**Net carb**	**4.2 g**

Vanilla Pecan Bars

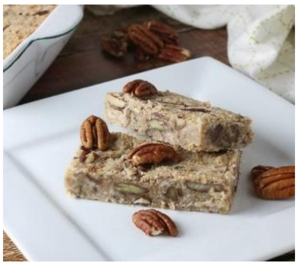

Makes 4 serves (4 bars) total; 1 serve = 1 bar.
Preparation time: VERY LONG (>45 minutes; but nearly all oven and fridge time).

Dry Ingredients:
1 cup pecan (or walnut) halves
1/2 cup almond meal
1/4 cup ground flaxseed
1 tbsp Natvia

Wet Ingredients:
5 tbsp butter (or extra virgin coconut oil), melted
2 tbsp pure vanilla extract

(1) Preheat the oven to 180 C. Place the pecans in a small plastic bag and crush with a rolling pin.
(2) Add the pecans and rest of the dry ingredients to a large bowl and mix together.
(3) Add the butter and vanilla to the dry ingredients. Mix into a crumbly dough.
(4) Press the dough into a small baking dish and bake 15-20 minutes.
(5) Remove the dish from the oven and let it cool for at least 15 minutes, then place it in the fridge for at least 1 hour (or overnight) to solidify. Cut into four equal-sized bars and serve.

PER SERVE:

**Calories**	**442**
**Fat**	**44.6 g (12.0 g saturated)**
**Protein**	**7.3 g**
**Fibre**	**5.3 g**
**Net carb**	**2.1 g**

Salads

Caprese Salad

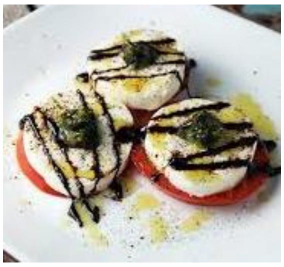

Makes 2 serves.
Preparation time: SHORT (<15 minutes).

Paste:
3 tbsp fresh basil, chopped
2 tbsp extra virgin olive oil

Salad:
3 tbsp extra virgin olive oil
1 tomato
125 g ball Massimo's mozzarella cheese
2 tsp balsamic vinegar
1/2 tsp salt, pepper to taste

(1) Add the basil leaves and 2 tbsp olive oil to a blender. Pulse until you have a basil paste, then set aside.
(2) Slice the tomato half into three thick slices, then do the same to the mozzarella ball. Arrange on a plate by placing each mozzarella slice on top of a tomato slice.
(3) When ready to eat, pour the paste over the top of the tomato and mozzarella, then pour the remaining olive oil on top. Drizzle over the vinegar, season with salt and pepper, and serve.

PER SERVE:

**Calories**	**507**
**Fat**	**46.4 g (12.2 g saturated)**
**Protein**	**16.9 g**
**Fibre**	**0.8 g**
**Net carb**	**5.9 g**

Strawberry Salad

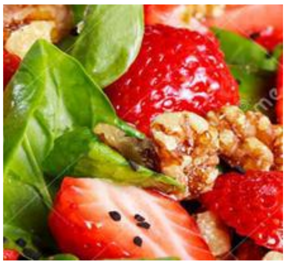

Makes 3 serves.
Preparation time: SHORT (<15 minutes).

Salad:
60 g (1/2 bag) spinach and kale leaves
1/2 cup strawberry halves
1 avocado
1/3 cup walnut (or pecan) halves
1/3 cup macadamia nuts

Dressing:
Juice from 1/2 lemon
1/3 cup extra virgin olive oil
1 tbsp apple cider vinegar

(1) Add the leaves, berries, sliced avocado, and nuts to a large bowl.
(2) Pour the lemon juice, oil, and vinegar over top. Toss and serve.

PER SERVE:

**Calories**	**533**
**Fat**	**55.3 g (7.6 g saturated)**
**Protein**	**4.6 g**
**Fibre**	**7.2 g**
**Net carb**	**4.3 g**

Mediterranean Salad

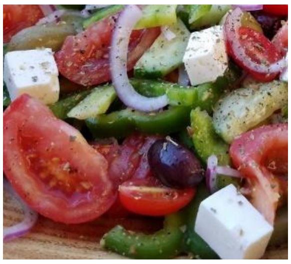

Makes 3 serves.
Preparation time: MODERATE (15-30 minutes).

Salad:
1 large (telegraph) cucumber
5 cherry tomatoes (or 1 tomato)
1/4 red onion
1/2 capsicum
100 g whole pitted olives
1/3 cup almonds
100 g goat or cow feta cheese
1 tbsp fresh basil, chopped

Dressing:
Juice from 1 lemon
1/2 cup extra virgin olive oil
1 tbsp balsamic vinegar
1 tsp salt, pepper to taste

(1) Slice and add the cucumber, tomatoes, onion, capsicum, olives, almonds (whole or slivered), feta cheese, and basil to a large bowl.
(2) Pour the lemon juice, oil, and vinegar over top. Sprinkle plenty of salt and pepper over the salad and toss to combine. Serve (eat all the oil).

PER SERVE:

**Calories**	**577**
**Fat**	**55.8 g (10.5 g saturated)**
**Protein**	**9.5 g**
**Fibre**	**4.4 g**
**Net carb**	**6.6 g**

Greek Salad

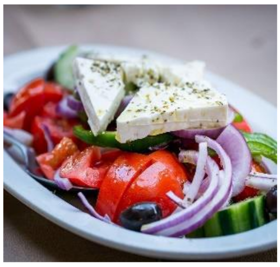

Makes 3 serves.
Preparation time: MODERATE (15-30 minutes).

Salad:
1 large (telegraph) cucumber
5 cherry tomatoes (or 1 tomato)
1/4 red onion
1/2 capsicum
100 g whole pitted olives
1 avocado
150 g goat or cow feta cheese

Dressing:
1/2 cup extra virgin olive oil
1 tbsp balsamic vinegar
1 tsp oregano
1 tsp salt, pepper to taste

(1) Combine sliced cucumber, tomatoes, onion, capsicum, and olives, in a salad bowl.
(2) Pour the oil and vinegar over the salad. Sprinkle over oregano and salt and pepper.
(3) Top with the sliced avocados and feta cheese. Serve (eat all the oil).

PER SERVE:

**Calories**	**637**
**Fat**	**60.9 g (13.5 g saturated)**
**Protein**	**9.9 g**
**Fibre**	**7.1 g**
**Net carb**	**6.5 g**

Broccoli & Bacon Salad

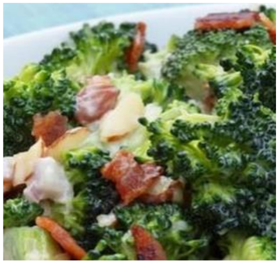

Makes 4 serves.
Preparation time: MODERATE (15-30 minutes).

Dressing:
1/3 cup extra virgin olive oil
1/3 cup traditional sour cream
Juice from 1 lemon

Salad:
1 tbsp extra virgin coconut oil (or butter)
8 strips streaky bacon
1½ tsp salt, pepper to taste
1 head broccoli
1/4 red onion
1/2 cup sliced almonds
100 g mozzarella (or your favourite) cheese, grated

(1) Combine the olive oil, sour cream, and lemon juice in a blender, once blended set the dressing aside in a small bowl.
(2) Heat the coconut oil in a pan over medium heat. Slice and fry the bacon strips, adding plenty of salt and pepper as they cook.
(3) Meanwhile, slice and add the broccoli, onion, and almonds to a large bowl. When the bacon is ready, add it to the bowl (along with all the oil) to create the salad.
(4) Pour the dressing over top of the salad, toss to combine, sprinkle over the grated cheese, and serve.

PER SERVE:

**Calories**	**619**
**Fat**	**58.0 g (18.4 g saturated)**
**Protein**	**19.9 g**
**Fibre**	**3.7 g**
**Net carb**	**5.6 g**

Smoked Salmon Salad

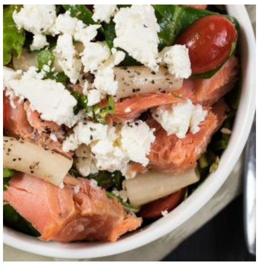

Makes 3 serves.
Preparation time: MODERATE (15-30 minutes).

Salad:
200 g smoked salmon
1 large (telegraph) cucumber
5 cherry tomatoes (or 1 tomato)
1/3 cup almonds (alternatively, use macadamia or Brazil nuts)
100 g goat or cow feta cheese
1-2 tbsp fresh basil, chopped

Dressing:
1/2 cup extra virgin olive oil
1-2 tbsp balsamic vinegar
1 tsp salt, 1 tsp pepper

(1) Slice and add the salmon, cucumber, tomatoes, almonds (whole or slivered), feta cheese, and basil to a large bowl.
(2) Pour over the olive oil and vinegar, sprinkle over salt and pepper, toss to combine, and serve (eat all the oil).

PER SERVE:

**Calories**	**607**
**Fat**	**54.1 g (11.2 g saturated)**
**Protein**	**21.1 g**
**Fibre**	**2.9 g**
**Net carb**	**8.1 g**

Basil Pesto Chicken Salad

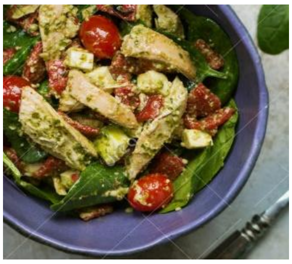

Makes 4 serves.
Preparation time: MODERATE (15-30 minutes).

Marinade:
1/3 cup basil pesto
1/2 cup extra virgin olive oil
2 tbsp balsamic vinegar

Salad:
500 g chicken thighs
60 g (1/2 bag) salad mix
1 avocado
5 cherry tomatoes (or 1 tomato)
2 tbsp fresh basil, chopped
1 tsp salt, pepper to taste
100 g mozzarella (or your favourite) cheese, grated

(1) To make the marinade, mix the pesto, oil, and vinegar in a large bowl.
(2) Slice the chicken thighs into bite-size strips and place them in the large bowl of marinade, turn to fully coat, then place in the fridge for 10-15 minutes.
(3) While the chicken is in the fridge, put the salad mix into another large bowl, followed by the sliced avocado, tomatoes, and basil.
(4) When the fridge time is done, set a large pan over medium heat. Place the chicken thighs and their marinade into the pan and cook for 6-7 minutes on each side of the thighs. Season with salt and pepper as you cook.
(5) Place the chicken and marinade on the salad followed by the grated cheese on top, and serve.

PER SERVE:

**Calories**	**648**
**Fat**	**54.2 g (11.7 g saturated)**
**Protein**	**30.8 g**
**Fibre**	**4.4 g**
**Net carb**	**6.7 g**

Stews & Soups

Garden Chicken

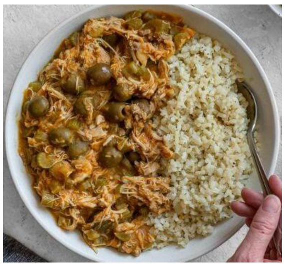

Makes 3 serves.
Preparation time (excluding crockpot time): SHORT (<15 minutes).

1/4 cup extra virgin olive oil
1/4 cup butter
1/2 brown onion
4 stalks celery
2 tsp oregano
500 g chicken thighs
1 tsp salt, pepper to taste
1/4 cup tomato puree
1/4 cup sundried tomato pesto
1 cup chicken stock
200 g green pitted olives
Juice from 1 lemon

(1) Slice the chicken and vegetables. Place everything in the crockpot.
(2) Mix the ingredients together, then set the crockpot on low for 6-7 hours (or high for 3-4 hours).
(3) Once cooked, place in a bowl and serve (goes well with traditional sour cream or cauliflower rice).

PER SERVE:

**Calories**	**642**
**Fat**	**54.5 g (17.0 g saturated)**
**Protein**	**32.5 g**
**Fibre**	**3.9 g**
**Net carb**	**4.9 g**

Rustic Beef Stew

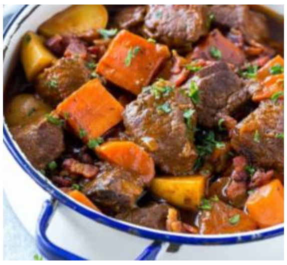

Makes 4 serves.
Preparation time (excluding crockpot time): SHORT (<15 minutes).

1/3 cup extra virgin olive oil
600 g beef (as high in fat as possible), lamb, or steak
200 g radishes
3 stalks celery
1 carrot
2 cups beef stock
1 tbsp whole grain or Dijon mustard
1 tbsp Worcester sauce
2 tsp (each) of garlic powder, rosemary, and thyme
2 tsp salt, pepper to taste
1/2 bunch fresh parsley (or coriander), chopped

(1) Heat the oil in a pan over low-medium (NOT high) heat. Slice the meat into 1-inch chunks and place in the pan until lightly browned all over.
(2) While the meat is browning in the pan, slice the radishes, celery, and carrot and set aside.
(3) Place all other ingredients into the crockpot, then mix together until well incorporated.
(4) Add the sliced meat (plus oil) and vegetables to the crockpot, then cook everything on low for 6-7 hours (or high for 3-4 hours).
(5) Mix in the parsley and serve (goes well with traditional sour cream or cauliflower rice).

PER SERVE:

**Calories**	**624**
**Fat**	**47.4 g (12.9 g saturated)**
**Protein**	**42.1 g**
**Fibre**	**1.5 g**
**Net carb**	**4.7 g**

Hamburger Soup

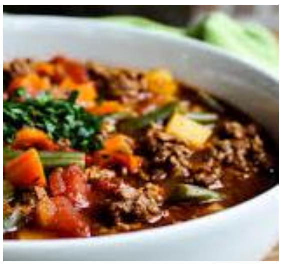

Makes 3 serves.
Preparation time: MODERATE (15-30 minutes).

1/4 cup extra virgin olive oil
500 g regular (18% fat) beef mince
1 carrot
4 stalks celery
1 zucchini
1/4 brown onion
2 cups beef stock
1 tomato
1 tbsp fresh basil, chopped
1½ tsp salt, pepper to taste

(1) Heat the oil in a large pot over low-medium (NOT high) heat. Saute the mince until completely browned.
(2) Slice and mix in the carrot, celery, zucchini, and onion. Brown the vegetables slightly.
(3) Pour in the stock, followed by the chopped tomato. Bring to a boil, then turn down the heat to maintain a strong simmer for 10-15 minutes, stirring occasionally.
(4) Season with the basil s followed by salt and pepper, then serve.

PER SERVE:

**Calories**	**651**
**Fat**	**47.4 g (13.6 g saturated)**
**Protein**	**47.0 g**
**Fibre**	**1.9 g**
**Net carb**	**6.7 g**

Thai Chicken Soup

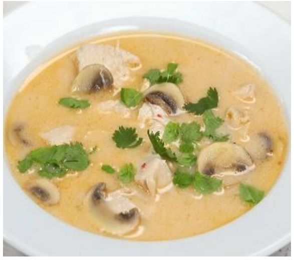

Makes 2 serves.
Preparation time: LONG (30-45 minutes).

2 cups chicken stock
2 tbsp cold pressed extra virgin coconut oil
2 stalks spring onions
Juice from 1 lime
1/2 tsp salt
500 g chicken thighs
8-12 mixed mushrooms (depends on size)
1½ cups canned coconut cream
3 tbsp fresh coriander, chopped

(1) Heat the stock and coconut oil in a large pot over medium-high heat. Add the sliced spring onions as well as the lime juice, ginger, and salt. Reduce the heat and simmer 5 minutes.
(2) Add the whole chicken thighs and sliced mushrooms. Simmer 15-20 minutes.
(3) Remove the chicken thighs and shred them, then add them back in along with the coconut cream. Simmer 5 minutes; taste and add more salt if needed.
(4) Garnish with the coriander and serve.

PER SERVE:

**Calories**	**620**
**Fat**	**43.6 g (29.1 g saturated)**
**Protein**	**49.9 g**
**Fibre**	**3.4 g**
**Net carb**	**5.4**

Burgers & Breads

Feta Burgers

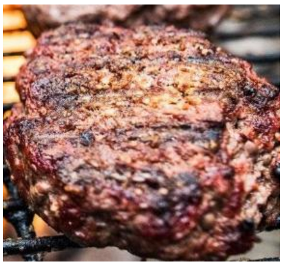

Makes 3 serves (3 burgers) total; 1 serve = 1 burger.
Preparation time: SHORT (<15 minutes).

500 g regular (18% fat) beef mince
1 tsp Worcester sauce
1 tsp steak seasoning
1/2 tsp salt, pepper to taste
100 g feta cheese
2 tbsp butter (or extra virgin coconut oil)

(1) Knead together the mince, Worcester sauce, seasoning, salt, and pepper in a bowl. Divide it out to create six equal-sized patties. Flatten to make them thin.
(2) Top three patties with the crumbled feta cheese (one-third of the cheese on each), but leave the other three patties bare.
(3) Place a bare patty on top of a cheese-topped patty to make a burger. Press the edges together to seal in the cheese (seal them well). Do this to until you have three burgers ready to cook.
(4) Heat the butter in a large pan over medium-high heat. Place the patties in the butter and cook 5-7 minutes on each side (make sure patties are fully cooked on the inside). Remove from heat, drip some of the butter over the burgers, and serve (goes well with traditional sour cream, mayonnaise, or aioli).

PER SERVE:

**Calories**	**614**
**Fat**	**44.9 g (21.3 g saturated)**
**Protein**	**49.2 g**
**Fibre**	**0 g**
**Net carb**	**0.5 g**

Cheeseburgers

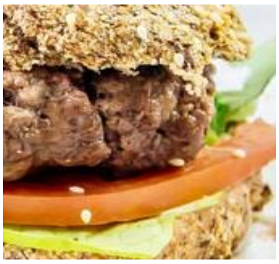

Makes 4 serves (4 burgers) total; 1 serve = 1 burger.
Preparation time: MODERATE (15-30 minutes).

1/3 cup traditional sour cream (alternatively, can use mayonnaise or aioli)
1 tsp Worcester sauce
500 g regular (18% fat) beef mince
1 tsp apple cider vinegar
1½ tsp salt, pepper to taste
2 tbsp butter
4 large lettuce leaves (or 8 keto tortillas)
4 slices mozzarella (or your favourite) cheese
1 tomato

(1) Mix together the sour cream and Worcester sauce in a bowl. Set aside.
(2) Knead together the mince, vinegar, salt, and pepper in a bowl. Create four equal-sized patties (1-2 cm thick). Pierce each patty several times with a fork.
(3) Heat the butter in a large pan over medium-high heat. Place the patties in the sizzling butter and cook 4-5 minutes, then flip them and cook another 3-4 minutes (make sure patties are fully cooked on the inside).
(4) When done, transfer each patty to a leaf, and quickly top each patty with a cheese slice so the cheese melts a bit. Add sliced tomato, 1½ tbsp of the sour cream and Worcester sauce, and more salt and pepper, then close up the leaves to make four burgers and serve.

PER SERVE:

**Calories**	**519**
**Fat**	**38.0 g (19.6 g saturated)**
**Protein**	**39.5 g**
**Fibre**	**0.6 g**
**Net carb**	**2.3 g**

Chicken Sandwiches

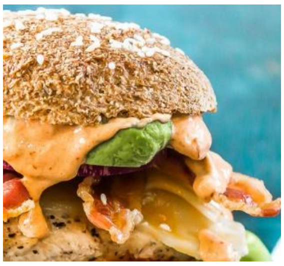

Makes 4 serves (4 big sandwiches) total; 1 serve = 1 sandwich.
Preparation time: MODERATE (15-30 minutes).

1/3 cup traditional sour cream (alternatively, can use mayonnaise or aioli)
1 tsp Worcester sauce
2 tbsp butter
500 g (should be 4) chicken thighs
4 strips streaky bacon
1½ tsp salt, pepper to taste
4 large lettuce leaves (or 8 keto tortillas)
4 slices mozzarella (or your favourite) cheese
1 avocado

(1) Mix together the sour cream and Worcester sauce in a bowl. Set aside.
(2) Heat the butter in a large pan over medium heat, then fry the thighs (leave them whole) and bacon strips, adding the salt and pepper as they cook.
(3) When done, transfer each thigh to a leaf and quickly top each thigh with a cheese slice so the cheese melts a bit. Add bacon, sliced avocado, 1½ tbsp of the sour cream and Worcester sauce, and more salt and pepper, then close up the leaves to make four sandwiches and serve.

PER SERVE:

**Calories**	**490**
**Fat**	**37.7 g (18.0 g saturated)**
**Protein**	**33.3 g**
**Fibre**	**3.7 g**
**Net carb**	**2.7 g**

Keto Tortillas

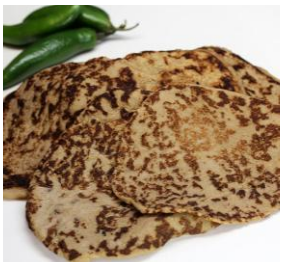

Makes 8 serves (8 tortillas) total; 1 serve = 1 tortilla.
Preparation time: LONG (30-45 minutes).

1 cup coconut flour
1/4 cup psyllium husk
1/2 tsp (each) of garlic powder and cumin powder
1/2 cup butter, melted
2 cups hot water
2 tbsp extra virgin coconut oil (or butter)

(1) Add all dry ingredients to a large bowl and mix together.
(2) Mix in all the butter, then mix in the water until well incorporated.
(3) After it cools, roll the dough into a log shape and cut into eight equal parts, then flatten each part into a circular tortilla (if you want a perfect circle, after flattening the dough punch out a circle using a small pan lid).
(4) Melt the coconut oil in a pan over medium heat and cook each tortilla until both sides are brown; you may need to add more oil if the pan dries up. Remove and serve (versatile recipe that goes well with many recipes in this program, but it also goes well on its own with butter or traditional sour cream).

PER SERVE:

**Calories**	**323**
**Fat**	**20.5 g (15.6 g saturated)**
**Protein**	**6.9 g**
**Fibre**	**20.8 g**
**Net carb**	**3.0 g**

Keto Crackers

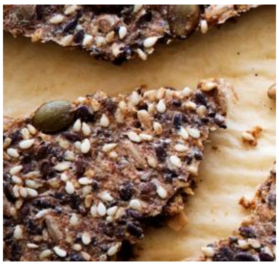

Makes 16 serves (16 big crackers) total; 1 serve = 1 cracker.
Preparation time: VERY LONG (>45 minutes; but nearly all oven time).

2/3 cup sunflower seeds
2/3 cup pumpkin seeds
1/2 cup sesame seeds
1/2 cup flax seeds
3 tbsp psyllium husk
1/4 cup almond meal
2 tbsp coconut flour
1¼ cups water
3 tbsp butter, melted
1½ tsp salt

(1) Preheat oven to 150 C. Mix all ingredients in a large bowl and let sit for 10 minutes until the mixture thickens.
(2) Spread out thinly on two baking trays lined with wax paper, or greased with butter or coconut oil.
(3) Bake for 50-60 minutes. Cool and serve (goes well with butter or traditional sour cream).

PER SERVE:

**Calories**	**170**
**Fat**	**13.8 g (3.3 g saturated)**
**Protein**	**5.3 g**
**Fibre**	**6.9 g**
**Net carb**	**2.6 g**

Keto Bread

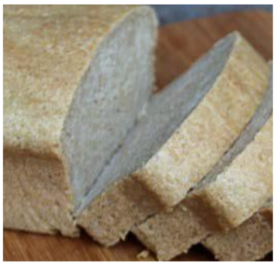

Makes 8 serves (8 buns or slices) total; 1 serve = 1 bun or slice.
Preparation time: VERY LONG (>45 minutes; but nearly all oven time…this is great bread but takes time to make it, you want to just buy bread instead, I recommend Home St. Keto Bread).

4 eggs
1½ cups cashews
1/4 cup butter
2 tbsp apple cider vinegar
1/4 cup pouring cream
1/4 cup coconut flour
1 tsp baking soda
1 tsp salt

(1) Place a small dish of water on the bottom oven rack and preheat oven to 175 C. Separate egg yolks from whites and place each in a separate bowl. Set both aside.
(2) Blend the cashews and butter in a processor or blender until a cashew butter is formed. Scrape the sides down as needed. Then add the yolks, vinegar, and cream to the cashew butter in the blender, and blend again until well mixed. Pour this new cashew butter mixture into a large bowl.
(3) Combine the coconut flour, baking soda, and salt in a small bowl, then combine with the cashew butter mixture in the large bowl.
(4) Beat the egg whites until stiff peaks form, then gently fold into the mixture in the large bowl to form the batter. Pour the batter into a loaf pan coated with butter and bake for 50 minutes.
(5) Once done, remove the bread and cool for 20 minutes before serving (goes well with butter or traditional sour cream). Store in plastic wrap in the fridge to prevent drying out.

PER SERVE:

**Calories**	**328**
**Fat**	**26.0 g (9.8 g saturated)**
**Protein**	**9.9 g**
**Fibre**	**5.5 g**
**Net carb**	**6.5 g**

Keto Nutloaf

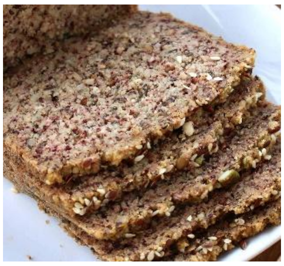

Makes 12 serves (12 slices) total; 1 serve = 1 slice.
Preparation time: VERY LONG (>45 minutes; but nearly all oven time).

Dry Ingredients:
1 cup almonds
1 cup ground flaxseed
1/2 cup cashews
1/2 cup pumpkin seeds
1 cup sunflower seeds
1/4 cup walnut halves
1/2 tbsp baking powder
2 tsp salt

Bind Ingredients:
1/2 cup chia seeds
3 cups warm water

(1) Preheat the oven to 180 C. Combine the dry ingredients together in a large bowl (however, set aside half the sunflower seeds for later).
(2) Blend the dry ingredients in a processor or blender until they are ground; you may need to do this in stages rather than all at once. Once done, place the ground mixture back in the large bowl with the whole sunflower seeds that you initially set aside.
(3) In a separate bowl, mix the bind ingredients into a paste. Add the paste to the large bowl with the dry ingredients and mix together. Add more water if required.
(4) Bake for 45 minutes in a loaf pan greased with butter. Serve warm (goes well with butter or traditional sour cream).

PER SERVE:

**Calories**	**294**
**Fat**	**23.9 g (2.8 g saturated)**
**Protein**	**10.0 g**
**Fibre**	**9.4 g**
**Net carb**	**4.6 g**

European

Slow-Cooked Lamb

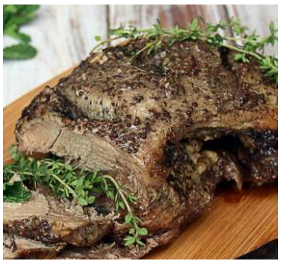

Makes 3 serves.
Preparation time (excluding crockpot time): SHORT (<15 minutes).

500 g lamb (leg or shank)
1 tbsp whole grain or Dijon mustard
1/2 tbsp maple syrup
1 tsp salt, pepper to taste
1 tsp (each) of dried rosemary and crushed garlic
1/4 cup extra virgin olive oil
1 tbsp thyme

(1) Place the lamb in a crockpot and cut three slits (3-4 cm deep) along the top of it.
(2) Rub the lamb with the mustard and syrup, sprinkle over salt and pepper, then insert as much rosemary and garlic as you can into each slit until used up.
(3) Pour olive oil all over the lamb, then set the crockpot to low and cook for 6 hours.
(4) Open the lid, sprinkle thyme over top, then cook for 1 hour more.
(5) Remove, cool, and serve (eat as much oil as you can).

PER SERVE:

**Calories**	**482**
**Fat**	**46.6 g (6.4 g saturated)**
**Protein**	**47.6 g**
**Fibre**	**0.2 g**
**Net carb**	**2.4 g**

Tuscan Salmon

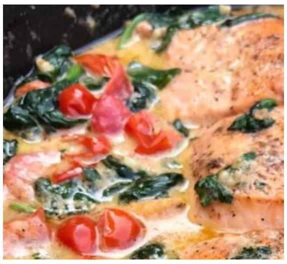

Makes 3 serves.
Preparation time: MODERATE (15-30 minutes).

3 tbsp extra virgin olive oil
400 g salmon fillets
1 tsp salt, pepper to taste
3 tbsp butter
3 cloves garlic (or 3 tsp crushed garlic)
5 cherry tomatoes (or 1 tomato)
60 g (1/2 bag) spinach (or kale) leaves
1/2 cup canned coconut cream
50 g parmesan cheese
1/4 cup fresh herbs (basil or parsley), chopped
Juice from 1 lemon

(1) Heat the olive oil in a pan over medium (NOT high) heat. Season salmon fillets with salt and pepper.
(2) Cook the salmon fillets 2-3 minutes on one side. Flip them over and cook 2-3 minutes on the other side (do not overcook).
(3) Melt the butter in the pan, add the minced garlic, and cook for 1 minute, then add in the sliced tomatoes, season with more salt and pepper, and cook another 2-3 minutes. Break up the fillets as you cook them. Add the spinach and cook until it begins to wilt.
(4) Stir in the coconut cream, sliced cheese, and herbs. Reduce heat to low and simmer 5 minutes, until the sauce is slightly reduced (mix it to prevent burning).
(5) Squeeze lemon juice over top before serving.

PER SERVE:

**Calories**	**585**
**Fat**	**48.2 g (17.7 g saturated)**
**Protein**	**34.4 g**
**Fibre**	**1.4 g**
**Net carb**	**1.9 g**

Caprese Chicken

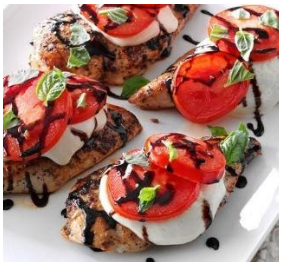

Makes 4 serves; 1 serve = 1 thigh.
Preparation time: MODERATE (15-30 minutes).

1/3 cup extra virgin olive oil
500 g chicken thighs
1/2 tsp salt, pepper to taste
125 g (or 4 slices) mozzarella cheese
5 cherry tomatoes (or 1 tomato)
2 tbsp balsamic vinegar
2 tbsp fresh basil, chopped

(1) Preheat oven to 190 C. Heat the olive oil in a large ovenproof pan over medium (NOT high) heat. Season the chicken with salt and pepper and cook in the oil, about 2-3 minutes per side.
(2) Transfer the thighs to the oven, bake 20 minutes, then remove from oven.
(3) Top the thighs with sliced cheese and tomatoes and bake 3 more minutes.
(4) Remove from oven, sprinkle over basil and vinegar, and place on a plate. Pour all the oil and vinegar from the pan over the thighs, and serve (eat all the oil).

PER SERVE:

**Calories**	**430**
**Fat**	**32.2 g (8.9 g saturated)**
**Protein**	**30.1 g**
**Fibre**	**0.3 g**
**Net carb**	**4.2 g**

Lemony Gurnard

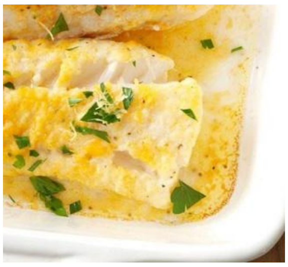

Makes 2 serves.
Preparation time: MODERATE (15-30 minutes).

1/4 cup almond meal
1/2 tbsp (each) of dill and chives
1 tsp (each) of onion powder and garlic powder
1/2 tsp salt, pepper to taste
400 grams fresh Gurnard (or any whitefish) fillets
1/3 cup butter (or extra virgin coconut oil)
Juice from 2 lemons

(1) Mix together the almond meal, all spices, and salt and pepper in a large bowl.
(2) Take the fish fillets, one at a time, and press into the flour mix. Turn and repeat. You want to really cover them well and place on a separate plate once done. Use all the flour mix.
(3) In a large pan, heat the butter and lemon juice over medium-high heat. You want it hot enough to crust the flour mix but not so hot to burn the butter.
(4) Fry the fish fillets 4-5 minutes, flip, and fry another 2-3 minutes. Wriggle the pan frequently so the fillets soak up the butter and juice. Don't let your pan dry out; add more butter if necessary. The fillet coating should be golden brown when done.
(5) Check if the fish fillets are done with a fork and remove from the pan when almost done (do not overcook), pour all the buttery sauce over them, and serve (eat all the buttery sauce).

PER SERVE:

**Calories**	**626**
**Fat**	**52.9 g (24.9 g saturated)**
**Protein**	**37.5 g**
**Fibre**	**0.5 g**
**Net carb**	**0.9 g**

Keto Pizza

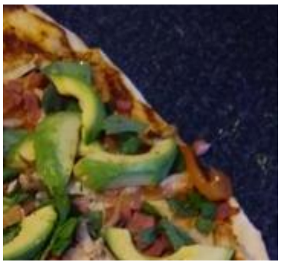

Makes 6 serves.
Preparation time: MODERATE (15-30 minutes).

1¾ cups almond meal
2 tbsp coconut flour
1/2 tsp (each) of xanthum gum and baking powder
Pinch (each) of salt, garlic powder, and onion powder
2 eggs
2 tbsp butter, melted
1/2 cup crushed tomatoes
120 g mozzarella cheese
200 g Italian salami (or smoked salmon)
2 mushrooms
75 g whole pitted olives

(1) Preheat the oven to 200 C. Mix all the dry ingredients into a large bowl, then add the eggs and butter and mix until combined. Roll into a ball of dough with your hands.
(2) Grease a baking tray (or shallow baking dish) with some butter, then flatten the dough into the tray, aiming to make it 25-30 cm in diameter. Bake 10 minutes then remove from oven.
(3) Place the tomatoes and cheese over top, and bake another 7-8 minutes. Remove from oven.
(4) Place the sliced mushrooms and olives over top, and bake another 2-3 minutes. Remove from oven and serve.

PER SERVE:

**Calories**	**482**
**Fat**	**39.9 g (11.7 g saturated)**
**Protein**	**20.1 g**
**Fibre**	**4.6 g**
**Net carb**	**4.7 g**

Salmon Fry

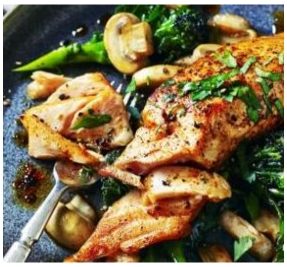

Makes 2 serves.
Preparation time: MODERATE (15-30 minutes).

3 mushrooms
1/2 capsicum
2 stalks spring onions
2 tbsp extra virgin olive oil
400 g salmon fillets
1 tsp (each) of oregano and cumin seeds
1/2 tsp salt, pepper to taste
Juice of 1/2 lemon
2 tbsp butter (or extra virgin coconut oil)
2 tbsp pumpkin seeds
60 g (1/2 bag) spinach (or kale) leaves

(1) Slice the mushrooms, capsicum, and spring onions. Set aside.
(2) Heat the olive oil in a pan over medium (NOT high) heat. Generously season salmon fillets with seasonings, salt, and pepper. Cook the fillets for 3-4 minutes on one side, flip them over, and cook the other side until the middle is pink (do not overcook).
(3) Drip lemon juice over the fillets and transfer them to a plate, but leave the oil in the pan. Place another plate on top of the fillets to keep them warm while you make the vegetables.
(4) Turn heat to high and melt the butter, then add the mushrooms, capsicum, spring onion, and pumpkin seeds and cook for 2-3 minutes. Turn off the heat and add the spinach leaves; after 2-3 minutes, stir in the leaves (they should be slightly wilted).
(5) Sprinkle more salt and pepper over the vegetables and transfer them to the plate with the fillets. Pour any remaining buttery sauce over top and serve.

PER SERVE:

**Calories**	**652**
**Fat**	**51.0 g (14.7 g saturated)**
**Protein**	**43.7 g**
**Fibre**	**1.8 g**
**Net carb**	**3.2 g**

Greek Chicken

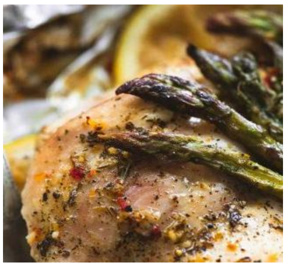

Makes 2 serves.
Preparation time: LONG (30-45 minutes; but nearly all fridge and oven time).

3 tbsp extra virgin olive oil
1 lemon
3 cloves garlic (or 3 tsp crushed garlic)
1-2 tsp oregano
3 tbsp butter (or extra virgin coconut oil)
500 g chicken thighs
1/2 tsp salt, pepper to taste
1 bunch asparagus
1 zucchini

(1) Preheat the oven to 200 C. In a large bowl, combine olive oil, juice from half the lemon, garlic, and oregano; whisk to combine. Add the chicken thighs, turn to fully coat them, and marinate in the fridge for 10-15 minutes.
(2) Heat the butter in a large oven-proof pan over medium-high heat. Remove the thighs from the fridge, season with salt and pepper, and add the chicken and marinade to the pan. Cook for 10 minutes.
(3) Flip chicken over and add the sliced asparagus, zucchini, and juice from the remaining half lemon. Cook another 2-3 minutes.
(4) Transfer to the oven and bake until the chicken is cooked through and vegetables are tender, usually about 15 minutes. Serve (eat all the buttery sauce).

PER SERVE:

**Calories**	**656**
**Fat**	**50.3 g (18.8 g saturated)**
**Protein**	**47.9 g**
**Fibre**	**2.5 g**
**Net carb**	**3.2 g**

Pan-American

Salsa Salmon

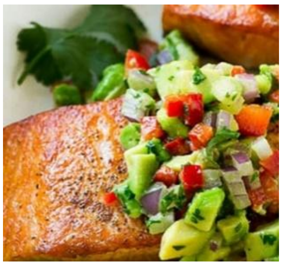

Makes 2 serves.
Preparation time: SHORT (<15 minutes).

Salsa:
1 avocado
1/4 red onion
1 tbsp fresh coriander, chopped
Juice from 2 limes

Salmon:
2 tbsp butter (or extra virgin coconut oil)
400 g salmon fillets
1 tsp (each) of cumin seeds, paprika, and onion powder
1/2 tsp salt, pepper to taste

(1) Mix together the sliced avocado, onion, and coriander in a bowl, then add lime juice and stir everything well to create the salsa. Place salsa in the fridge while you prepare the salmon.
(2) Melt the butter in a pan over medium heat. Season the salmon fillets with the spices, salt, and pepper and place in the pan.
(3) Cook the fillets for 3-4 minutes on one side. Flip them over and cook the other side until the middle is pink (do not overcook).
(4) Remove fillets from the pan onto a plate, top with the butter and salsa, and serve.

PER SERVE:

**Calories**	**632**
**Fat**	**48.0 g (13.9 g saturated)**
**Protein**	**42.0 g**
**Fibre**	**6.9 g**
**Net carb**	**2.5 g**

Bacon Avocado Wedges

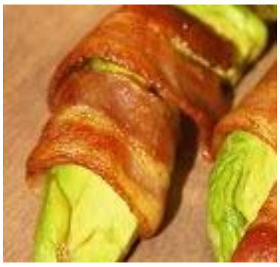

Makes 4 serves (12 wedges) total; 1 serve = 3 wedges.
Preparation time: MODERATE (15-30 minutes).

3 avocados
12 strips streaky bacon
1/4 cup traditional sour cream (alternatively, can use mayonnaise or aioli)

(1) Preheat oven to 200 C. Slice each avocado into four equally sized wedges.
(2) Wrap each wedge in bacon and place on a baking sheet.
(3) Bake 12-15 minutes, until the bacon is the way you want it.
(4) Top each wedge with 1 tsp of sour cream and serve.

PER SERVE:

**Calories**	**530**
**Fat**	**48.7 g (14.8 g saturated)**
**Protein**	**16.1 g**
**Fibre**	**10.1 g**
**Net carb**	**3.6 g**

Worcester Steaks

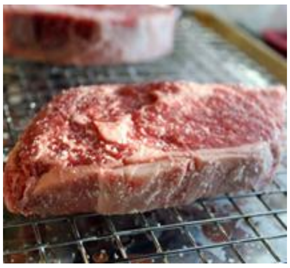

Makes 2 serves; 1 serve = 1 steak (could be more or less, depends on thickness).
Preparation time: LONG (30-45 minutes; but nearly all oven time).

400 g scotch fillet (or rib eye) steaks
1/2 tsp salt, pepper to taste
1/4 cup butter (alternatively, use animal fat or extra virgin coconut oil)
1 tbsp Worcester sauce
1 tbsp pouring cream

(1) Preheat oven to 125 C. Put the steaks on a wire rack over top of a cookie sheet (do not remove the fat). Season with salt and pepper and place in oven.
(2) Bake for 30 minutes, then turn the steaks over, season again, and bake another 10-20 minutes, depending how well you like your steaks done. Remove from oven.
(3) Heat the butter in a large pan over high heat. Wait until the butter is hot, then sear the steaks for 1-2 minutes on each side.
(4) At the end, drizzle Worcester sauce and cream over the steaks, flip to cover in sauce, and place on a plate. Pour all the buttery sauce from the pan over the steaks, and serve (eat all the sauce).

PER SERVE:

**Calories**	**692.9**
**Fat**	**50.5 g (26.3 g saturated)**
**Protein**	**59.3 g**
**Fibre**	**0 g**
**Net carb**	**1.7 g**

Keto Enchiladas

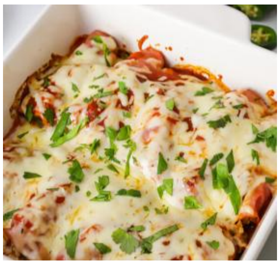

Makes 4 serves.
Preparation time: LONG (30-45 minutes).

3 tbsp butter (or extra virgin coconut oil)
500 g regular (18% fat) beef mince
1 tsp (each) of onion and garlic powder
2 tsp salt, pepper to taste
1/2 cup pre-made salsa
200 g sliced deli ham (8-12 slices depending on size)
100 g Monterey Jack (or your favourite) cheese

(1) Preheat the oven to 190 C. Heat the butter in a large pan over medium-high heat.
(2) Add the mince to the pan, breaking up the meat into small chunks as it browns. Mix in spices, salt, and pepper. Cook until thoroughly browned.
(3) Spread the salsa over the bottom of a baking dish. Lay the ham slices on a plate and place 1-2 tbsp of the meat mixture in the middle of each, then wrap the ham around the mixture. Place the ham wraps along the top of the salsa (squeeze them together as best you can).
(4) Place any leftover meat on top of the ham wraps, then sprinkle grated cheese over top.
(5) Place in the oven, bake 15-20 minutes, and serve (goes well with traditional sour cream).

PER SERVE:

Calories	582
Fat	40.2 g (18.5 g saturated)
Protein	47.7 g
Fibre	0.7 g
Net carb	3.9 g

Chicken Drumsticks

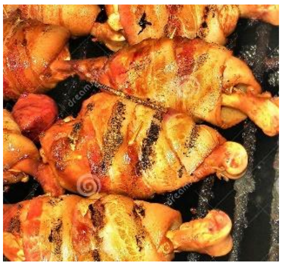

Makes 2 serves; 1 serve = 2 drumsticks.
Preparation time: LONG (30-45 minutes; but nearly all oven time).

4 chicken drumsticks (skin on)
4 strips streaky bacon
1/2 tsp salt, pepper to taste

(1) Preheat oven to 200 C. Line a baking tray with aluminium foil.
(2) Wrap one slice of bacon around each drumstick, working from the bottom of the drumstick to the top. Place on the baking tray and season with salt and pepper.
(3) Bake 45 minutes and serve (goes well with traditional sour cream, mayonnaise, or aioli).

PER SERVE:

**Calories**	**638**
**Fat**	**39.7 g (12.8 g saturated)**
**Protein**	**66.9 g**
**Fibre**	**0 g**
**Net carb**	**0.2 g**

Steak Fajitas

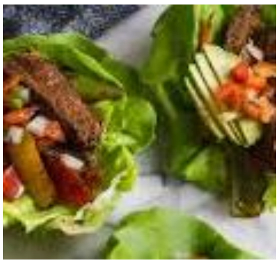

Makes 4 serves.
Preparation time: LONG (30-45 minutes).

3 tbsp butter (or extra virgin coconut oil)
2 capsicums (any colour)
1/4 red onion
400 g scotch fillet steaks
1 tsp salt, pepper to taste
4 large lettuce leaves (or keto tortillas)
1/4 cup traditional sour cream

(1) Heat a large pan over medium heat, then melt 2 tbsp butter in the pan. Slice up capsicums and onion, then add to the pan. You will cook all the vegetables for 12-15 minutes, but proceed to the next step when they start cooking.
(2) Heat another pan over medium-high heat, then melt 1 tbsp butter in that pan. Season steaks with salt and pepper, then cook each side for 4-5 minutes, until seared.
(3) Remove both pans from heat. Let steaks cool for 5 minutes, then slice into small chunks and mix in with the vegetables.
(4) Divide the vegetable steak mixture into 4 equal-sized portions. Place each portion on a lettuce leaf, top each portion with 1 tbsp sour cream, then wrap up the leaf and serve (can get a little bit messy, don't worry about it).

PER SERVE:

**Calories**	**355**
**Fat**	**24.2 g (12.6 g saturated)**
**Protein**	**30.8g**
**Fibre**	**1.6 g**
**Net carb**	**3.9 g**

Roast Pork

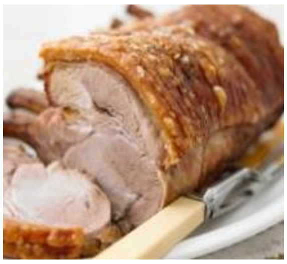

Makes 6 serves.
Preparation time: VERY LONG (>45 minutes; but nearly all oven time).

1 kg pork loin
2 tsp salt, pepper to taste
2 tsp thyme
3 cloves garlic (or 3 tsp crushed garlic)
2 tbsp whole grain or Dijon mustard
1½ cups traditional sour cream

(1) Place the pork loin fat side up in a roasting pan or baking dish small enough to hold it snugly. Season with salt and pepper. Let sit for 1 hour at room temperature.
(2) Preheat the oven to 240 C. Combine the thyme, garlic, and mustard in a small bowl. Using a pastry brush, coat the pork loin with the mustard mixture.
(3) Roast the pork loin 15 minutes, then remove from the oven. Turn the oven down to 95 C. Cover the pork loin with aluminium foil, then return to the oven and slow-roast for 8 hours.
(4) Remove the pork loin from the oven, slice thinly, and serve with generous amounts of sour cream on the side (1/4 cup sour cream per serve).

PER SERVE:

**Calories**	**551**
**Fat**	**37.6 g (16.5 g saturated)**
**Protein**	**47.6 g**
**Fibre**	**0.2 g**
**Net carb**	**2.6 g**

Asian

Palak Paneer

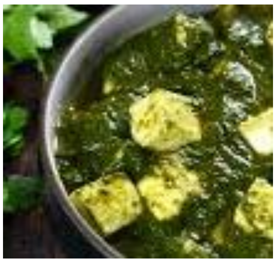

Makes 2 serves.
Preparation time: SHORT (<15 minutes).

3 tbsp butter (alternatively, can use extra virgin coconut oil or ghee)
1/2 brown onion
2 cloves garlic (or 2 tsp crushed garlic)
1 tsp cumin seeds
120 g (1 bag) spinach leaves
1/2 tsp salt
1/3 cup water
1/2 tsp (each) of turmeric, garam masala, coriander powder
1/3 cup canned coconut cream
200 g paneer (or haloumi) cheese

(1) Melt half the butter in a large pan over medium heat. Add the sliced onion, garlic, and cumin seeds and cook 2-3 minutes.
(2) Add spinach leaves and salt, then cook another 2-3 minutes. Once done, blend this spinach mixture with the water in a blender until a spinach puree forms, and set aside.
(3) Melt the other half of the butter in the pan. When melted, add the remaining spices and mix together.
(4) Pour in the spinach puree and cook 1-2 minutes, then add the cream and cubed paneer, cook for another 1-2 minutes, and serve (goes well with cauliflower rice).

PER SERVE:

**Calories**	**583**
**Fat**	**53.1 g (40.3 g saturated)**
**Protein**	**24.6 g**
**Fibre**	**2.4 g**
**Net carb**	**4.5 g**

Miti Fish

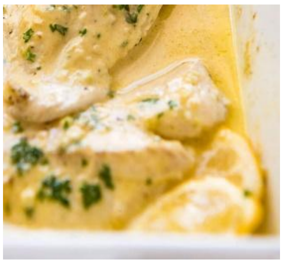

Makes 2 serves.
Preparation time: MODERATE (15-30 minutes).

1½ cups canned coconut cream
2 tbsp extra virgin coconut oil, melted
Juice of 1 lemon
1-2 chilli, finely grated (optional)
4 stalks spring onions, finely sliced
1/2 tsp salt
1/2 head broccoli
300 g whitefish

(1) Preheat oven to 200 C. In a bowl, combine all ingredients (except the fish) to make the sauce.
(2) Grease a baking pan with coconut oil. Place the fish in the pan, pour the sauce over top, bake 20 minutes or until fish is done, and serve.

PER SERVE:

**Calories**	**477**
**Fat**	**38.1 g (26.1 g saturated)**
**Protein**	**37.7 g**
**Fibre**	**4.3 g**
**Net carb**	**3.3 g**

Chicken Korma

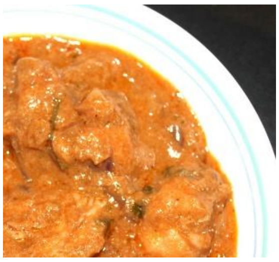

Makes 2 serves.
Preparation time: MODERATE (15-30 minutes).

500 g chicken thighs
1/4 cup plain Raglan/Cathedral Cove coconut yogurt
2 cloves garlic (or 2 tsp crushed garlic)
3 tbsp butter (alternatively, can use extra virgin coconut oil or ghee)
1 tsp (each) of curry powder and turmeric
1/2 tsp salt, pepper to taste
1/4 brown onion
1/2 cup chicken stock
1 cup canned coconut cream
1 small (Lebanese) cucumber

(1) Chop up the chicken into small chunks, mix it in a bowl with the yogurt and minced garlic, and marinate as you prepare the rest.
(2) Heat the butter in a large pan over low heat. Once melted, mix in the curry, turmeric, and salt and pepper. Stir intermittently from here on so that the spices don't stick to the pan.
(3) Turn heat to medium, then add the chicken marinade and onion. Cook 7-8 minutes, until no raw chicken bits are showing.
(4) Stir in the stock and cream, then simmer 7-8 minutes.
(5) Stir in the sliced cucumber, simmer 3-4 minutes, and serve (goes well with cauliflower rice).

PER SERVE:

Calories	672
Fat	50.7 g (36.4 g saturated)
Protein	47.9 g
Fibre	2.6 g
Net carb	5.5 g

Beef & Cashew Stirfry

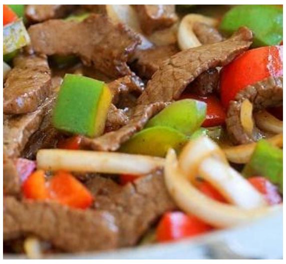

Makes 3 serves.
Preparation time: MODERATE (15-30 minutes).

1/4 cup extra virgin coconut oil
2 cloves garlic (or 2 tsp crushed garlic)
1 carrot
1/4 brown onion
1 zucchini
1/2 tsp salt, pepper to taste
400 g diced beef
1 tbsp of your favourite spices
1/4 cup beef stock
1/2 cup canned coconut cream
1/4 cup cashews
2 tbsp fresh basil, chopped

(1) Heat half the coconut oil in a wok over medium heat. Slice garlic, carrot, and onion, place in the wok, and cook until fragrant.
(2) Mix in the zucchini and salt and pepper, toss everything to combine, and set aside on a plate.
(3) Heat the rest of the coconut oil in a pan over medium heat and brown the beef slices on both sides. Season with your favourite spices.
(4) Add the vegetables back to the pan with the browned beef and mix everything together. Pour in the beef stock and coconut cream, then add the cashews and cook uncovered for 8 minutes.
(5) Lastly, add the basil and cook another 5 minutes before serving.

PER SERVE:

**Calories**	**573**
**Fat**	**48.1 g (27.3 g saturated)**
**Protein**	**28.1 g**
**Fibre**	**2.0 g**
**Net carb**	**5.7 g**

Paneer Makhanwala

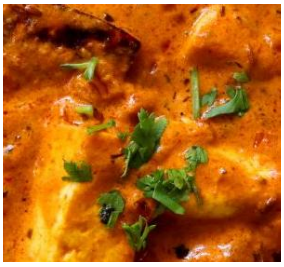

Makes 2 serves.
Preparation time: MODERATE (15-30 minutes).

3 tbsp butter (alternatively, can use extra virgin coconut oil or ghee)
1/4 brown onion
1 tsp cumin seeds
1 tsp (each) of turmeric and coriander
1/2 tsp salt
2 tbsp tomato puree
1/2 cup water
200 g paneer (or haloumi) cheese
1/3 cup canned coconut cream
2 tbsp fresh coriander, chopped

(1) Heat a large pan over medium and melt half the butter. Add the onion and cumin seeds and cook until the onion is translucent.
(2) Mix in the rest of the spices and salt, and cook for 2 minutes. Then add the tomato puree and water, lower the heat, and simmer 7-8 minutes.
(3) While the sauce is simmering, melt the remaining half of the butter in another pan, and fry the cubed cheese until golden brown.
(4) Mix in the cheese (and its buttery sauce), cream, and coriander to the pan with the simmering onion and spices, simmer another 1-2 minutes, and serve (goes well with cauliflower rice).

PER SERVE:

**Calories**	**578**
**Fat**	**52.8 g (40.3 g saturated)**
**Protein**	**23.1 g**
**Fibre**	**1.8 g**
**Net carb**	**5.1 g**

Thai Fish Curry

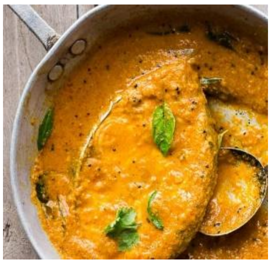

Makes 3 serves.
Preparation time: MODERATE (15-30 minutes).

1 tbsp extra virgin coconut oil
400 grams whitefish
1 tsp salt, pepper to taste
1/4 cup butter (or ghee)
1½ cups canned coconut cream
2 tbsp red curry paste
1/2 cup fresh coriander, chopped
1/2 head cauliflower (or broccoli)

(1) Preheat oven to 200 C. Grease a medium-sized baking dish with the coconut oil.
(2) Place 2-4 fish pieces (depends on size) snuggly in the baking dish. Salt and pepper generously and place 1-2 tbsp of butter on top of each fish piece.
(3) Mix the coconut cream, curry paste, and coriander in a small bowl and pour this over the fish. Bake in the oven 20 minutes, or until the fish is done.
(4) In the meantime, cut the cauliflower or broccoli into small florets, boil in lightly salted water for a couple of minutes, and serve with the fish (eat all the creamy curry).

PER SERVE:

**Calories**	**557**
**Fat**	**45.7 g (24.6 g saturated)**
**Protein**	**30.0 g**
**Fibre**	**4.8 g**
**Net carb**	**2.2 g**

Sticky Chicken Stirfry

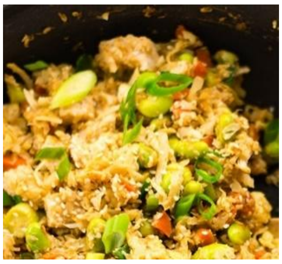

Makes 2 serves.
Preparation time: MODERATE (15-30 minutes).

500 g chicken thighs
1 tbsp Worcester sauce
1 cup grated cauliflower
3 mushrooms
3 stalks spring onion
1/2 capsicum
1/4 cup extra virgin coconut oil
1 tbsp almond (or peanut) butter
1/4 cup plain Raglan/Cathedral Cove coconut yogurt
1/2 tsp salt, pepper to taste
2 tsp (each) of cumin seeds, coriander, and curry powder
120 g (1 bag) spinach (or kale) leaves

(1) Slice the chicken thighs and marinate in the Worcester sauce while you prepare the cauliflower rice and vegetables.
(2) Grate the cauliflower into a large bowl, followed by the sliced mushrooms, spring onions, and capsicum; mix together and set aside.
(3) Melt the coconut oil in a large pan over medium heat, add the thighs and Worcester sauce, fry for 10-12 minutes, then add the cauliflower rice and vegetables and fry another 2-3 minutes.
(4) Mix in the almond butter, coconut yogurt, salt, pepper, and spices, then fry for 2-3 minutes or until everything is melted and mixed well.
(5) Finally, add the spinach and fry for a final 2-3 minutes. Mix in the spinach, and serve.

PER SERVE:

**Calories**	**691**
**Fat**	**50.8 g (34.6 g saturated)**
**Protein**	**50.5 g**
**Fibre**	**3.8 g**
**Net carb**	**8.6 g**

Lamb Curry

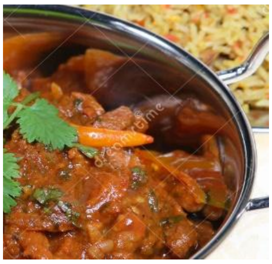

Makes 3 serves.
Preparation time: MODERATE (15-30 minutes).

3 tbsp butter (alternatively, can use extra virgin coconut oil or ghee)
400 g diced lamb
1/4 brown onion
2 stalks celery
2 cloves garlic (or 2 tsp crushed garlic)
1 tsp (each) of garam masala and turmeric
1 carrot
1½ cups canned coconut cream
1/4 cup water
1 tsp salt
Juice from 1/2 lemon
3 tbsp fresh coriander, chopped

(1) Melt the butter in a large pan over medium-high heat. Add the lamb and stir until slightly browned, about 5 minutes.
(2) Add the sliced onion and celery and cook another 2-3 minutes, until vegetables are soft. Bring the heat down to medium. Add the minced garlic and spices, cook another 2-3 minutes, until the lamb is cooked through.
(3) Add the sliced carrot, coconut cream, water, and salt. Bring to a simmer, and cook for 10-12 minutes.
(4) Sprinkle with lemon juice and coriander before serving (goes well with cauliflower rice).

PER SERVE:

**Calories**	**530**
**Fat**	**45.1 g (27.6 g saturated)**
**Protein**	**26.5 g**
**Fibre**	**2.6 g**
**Net carb**	**2.6 g**

Indian Eggplant

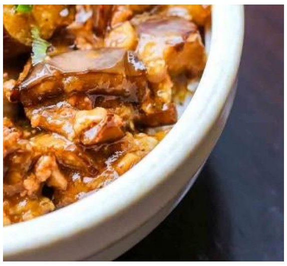

Makes 2 serves.
Preparation time: LONG (30-45 minutes; but nearly all oven time).

2 eggplants
1 tbsp extra virgin coconut oil
3 tbsp butter (alternatively, can use extra virgin coconut oil or ghee)
1 tsp cumin seeds
1/4 brown onion
2 cloves garlic (or 2 tsp crushed garlic)
1 tomato
1 tsp (each) of turmeric, garam masala, and paprika
1/2 tsp salt, pepper to taste
200 g paneer (or haloumi) cheese
2 tbsp fresh coriander, chopped

(1) Preheat oven to 180 C. Make four slits lengthwise in each eggplant and drizzle melted coconut oil over top to fully cover each. Place on a baking sheet and roast in the oven for 30-40 minutes, flipping halfway.
(2) While the eggplants are baking, add the butter to a pot over medium heat. Add the cumin and onion, then saute for 10 minutes.
(3) Add the sliced garlic, tomato, spices, salt and pepper, and cheese. Mix well. Cover the pan with a lid and cook 10-12 minutes, then remove the lid and fry another 2-3 minutes.
(4) The eggplant should be ready now. Remove from the oven and cool, remove the skin and chop the flesh. Stir the eggplant into the fry; cook another 5 minutes. Garnish with coriander and serve (goes well with cauliflower rice).

PER SERVE:

**Calories**	**684**
**Fat**	**56.6 g (43.6 g saturated)**
**Protein**	**26.4 g**
**Fibre**	**13.5 g**
**Net carb**	**12.5 g**

Veggie Sides

Marinated Olives

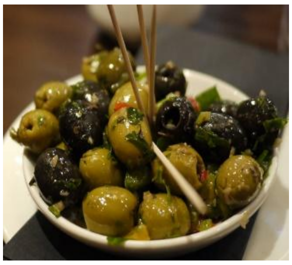

Makes 2 side-serves.
Preparation time: SHORT (<15 minutes).

200 g olives (seed-in is best)
Juice from 1/2 lemon
2 tbsp extra virgin olive oil
1 tsp coriander (optional)
Salt and pepper to taste

(1) Drain and place the olives in a bowl.
(2) Squeeze the lemon juice into a separate bowl, the add the olive oil followed by the coriander, salt, and pepper to create the dressing.
(3) Pour the dressing over the olives, let marinate for a while, and serve.

PER SERVE:

**Calories**	**267**
**Fat**	**28.8 g (3.3 g saturated)**
**Protein**	**1.1 g**
**Fibre**	**3.3 g**
**Net carb**	**1.3 g**

Cauliflower Rice

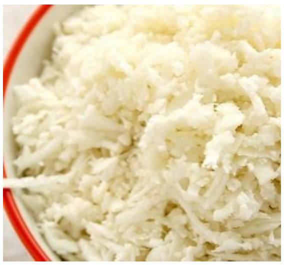

Makes 2 side-serves.
Preparation time: SHORT (<15 minutes).

1 cup grated cauliflower
2 tbsp butter (or extra virgin coconut oil)
1/4 tsp salt, pepper to taste
1/2 tsp (each) of ginger powder, coriander, garam masala, and cumin seeds (all optional)
2-3 tbsp fresh coriander, chopped (optional)

(1) Grate the cauliflower into a large bowl.
(2) Heat the coconut oil in a large pan over medium heat. Once melted, add the cauliflower and sprinkle salt over top. Cook 4-6 minutes.
(3) Stir in the spices if you are using it, then turn off the heat and add more salt if needed, then serve.

PER SIDE-SERVE:

**Calories**	**116**
**Fat**	**11.6 g (7.3 g saturated)**
**Protein**	**1.2 g**
**Fibre**	**1.4 g**
**Net carb**	**1.5 g**

Buttered Broccoli

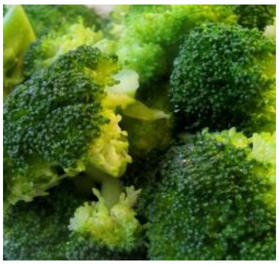

Makes 1 side-serve.
Preparation time: SHORT (<15 minutes).

1/2 head of broccoli
2 tbsp butter
1/4 tsp salt

(1) Boil some water in a pot.
(2) Chop the broccoli into florets and add them to the water.
(3) Cook until desired doneness is reached, anywhere from 1-4 minutes.
(4) Remove to a plate, add the butter and salt on top, and serve.

PER SIDE-SERVE:

**Calories**	**242**
**Fat**	**23.0 g (14.8 g saturated)**
**Protein**	**3.2 g**
**Fibre**	**3.0 g**
**Net carb**	**5.0 g**

Garlic Asparagus

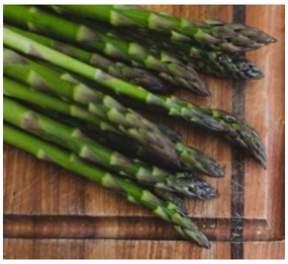

Makes 2 side-serves.
Preparation time: SHORT (<15 minutes).

1 bunch asparagus
2 tbsp butter (or extra virgin coconut oil)
2 cloves garlic (or 2 tsp crushed garlic)
1/4 tsp salt

(1) Wash the asparagus and separate the stalks.
(2) Boil some water in a pot and cook asparagus 2-3 minutes.
(3) Drain the asparagus and cool in cold water.
(4) Heat the butter and garlic in a pan over low-medium heat.
(5) Fry asparagus 2-3 minutes until browning, but be careful not to overcook. Add salt over top, and serve (eat all the buttery sauce).

PER SIDE-SERVE:

**Calories**	**120**
**Fat**	**11.6 g (7.3 g saturated)**
**Protein**	**2.1 g**
**Fibre**	**1.9 g**
**Net carb**	**1.7 g**

Worcester Brussels Sprouts

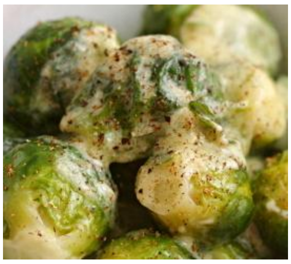

Makes 2 side-serves.
Preparation time: SHORT (<15 minutes).

1/3 cup butter (or extra virgin coconut oil)
10 Brussels sprouts
1/4 tsp salt, pepper to taste
1 tbsp Worcester sauce
1 tbsp pouring (or canned coconut) cream

(1) Place a frying pan over medium-high heat and add the oil.
(2) Wash the Brussels sprouts, cut them lengthwise, and add to the pan. Cook 3-4 minutes, or until sprouts are golden brown. Add salt and pepper.
(3) Pour the Worcester sauce and cream over the Brussels sprouts at the end, cook for another 30 seconds, and serve (eat all the buttery sauce).

PER SIDE-SERVE:

**Calories**	**380**
**Fat**	**37.6 g (23.8 g saturated)**
**Protein**	**3.9 g**
**Fibre**	**3.6 g**
**Net carb**	**6.6 g**

Vegetable Medley

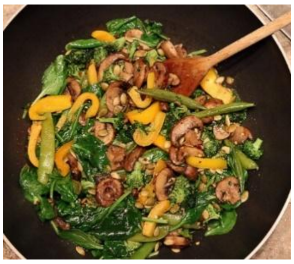

Makes 3 side-serves.
Preparation time: MODERATE (15-30 minutes).

2 tbsp butter (or extra virgin coconut oil)
2 cloves garlic (or 2 tsp crushed garlic)
4 mushrooms
1/2 head of broccoli
1/2 green capsicum
1/4 cup pumpkin seeds
1/2 tsp salt, pepper to taste
60 g (1/2 bag) spinach (or kale) leaves
1/4 cup extra virgin olive oil

(1) Prepare all the vegetables beforehand (this is important, it's so you can move quickly later) by slicing them into bite-size pieces. Make sure this is done before proceeding to the next step.
(2) Heat the butter in a pan on high heat. Once hot, add the minced garlic and mushrooms; make sure the mushrooms soak up as much oil as possible. Cook 1-2 minutes.
(3) In order, mix in the broccoli, capsicum, and pumpkin seeds. Season with salt and pepper. Cook another 1-2 minutes.
(4) Turn off the heat and add the spinach leaves. Wait 2-3 minutes, then pour the olive oil over top, stir in the leaves (they should be slightly wilted), and serve.

PER SIDE-SERVE:

**Calories**	**315**
**Fat**	**31.2 g (8.8 g saturated)**
**Protein**	**5.1 g**
**Fibre**	**2.4 g**
**Net carb**	**4.7 g**

Rukau

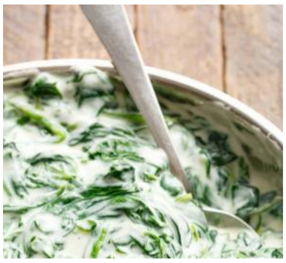

Makes 2 side-serves.
Preparation time: MODERATE (15-30 minutes).

1 tbsp butter (or extra virgin coconut oil)
1/2 brown onion
120 g (1 bag) spinach (or kale) leaves
1/4 tsp salt, pepper to taste
1½ cup coconut cream

(1) Bring a pan to medium heat on the stove, then melt the butter.
(2) Add the sliced onion to the pan and fry for 1-2 minutes.
(3) Add the spinach to the pan, cook until slightly wilted while adding salt and pepper.
(4) Add the coconut cream, turn heat to low and simmer 15-20 minutes, then serve.

PER SIDE-SERVE:

**Calories**	**249**
**Fat**	**23.1 g (15.7 g saturated)**
**Protein**	**4.2 g**
**Fibre**	**4.6 g**
**Net carb**	**4.1 g**

Dessert

Berries & Cream

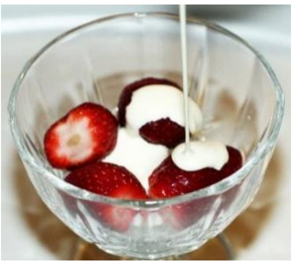

Makes 1 serve.
Preparation time: SHORT (<15 minutes).

3 strawberries <u>OR</u> 12 raspberries <u>OR</u> 12 blueberries
1/3 cup canned coconut cream (or pouring cream)

Preparation:
(1) Slice up the berries, if needed. Place in a small bowl.
(2) Mix in the cream and serve (eat all the cream).

PER SERVE:

**Calories**	**362**
**Fat**	**33.7 g (22.2 g saturated)**
**Protein**	**2.6 g**
**Fibre**	**0.9 g**
**Net carb**	**4.2 g**

MTP: Week 1 Meal Planner

Day	1st Meal Hour	2nd Meal Hour
Monday		
Tuesday		
Wednesday		
Thursday		
Friday		
Saturday		
Sunday		

To simplify things, make extra serves for the following day.

MTP: Week 1 Shopping List

**"Keto" Fruits (no fleshy fruits!)**<br>**(berries, avocados, olives etc)**	**Vegetables**<br>**(cauliflower, broccoli, capsicum etc)**
**Leafy Greens**<br>**(spinach, kale, lettuce etc)**	**Proteins**<br>**(meats, eggs, cheese etc)**
**Fats**<br>**(extra virgin olive oil, butter etc)**	**Pantry**<br>**(almond meal, ground flaxseed etc)**
**Nuts and Seeds**<br>**(almonds, walnuts, chia seeds etc)**	**Other**<br>**(whatever doesn't fit elsewhere)**

MTP: Week 2 Meal Planner

Day	1st Meal Hour	2nd Meal Hour
Monday		
Tuesday		
Wednesday		
Thursday		
Friday		
Saturday		
Sunday		

To simplify things, make extra serves for the following day.

MTP: Week 2 Shopping List

"Keto" Fruits (no fleshy fruits!) (berries, avocados, olives etc)	Vegetables (cauliflower, broccoli, capsicum etc)
**Leafy Greens** (spinach, kale, lettuce etc)	**Proteins** (meats, eggs, cheese etc)
**Fats** (extra virgin olive oil, butter etc)	**Pantry** (almond meal, ground flaxseed etc)
**Nuts and Seeds** (almonds, walnuts, chia seeds etc)	**Other** (whatever doesn't fit elsewhere)

MTP: Week 3 Meal Planner

Day	1st Meal Hour	2nd Meal Hour
Monday		
Tuesday		
Wednesday		
Thursday		
Friday		
Saturday		
Sunday		

To simplify things, make extra serves for the following day.

MTP: Week 3 Shopping List

“Keto” Fruits (no fleshy fruits!)<br>(berries, avocados, olives etc)	Vegetables<br>(cauliflower, broccoli, capsicum etc)
**Leafy Greens**<br>**(spinach, kale, lettuce etc)**	**Proteins**<br>**(meats, eggs, cheese etc)**
**Fats**<br>**(extra virgin olive oil, butter etc)**	**Pantry**<br>**(almond meal, ground flaxseed etc)**
**Nuts and Seeds**<br>**(almonds, walnuts, chia seeds etc)**	**Other**<br>**(whatever doesn’t fit elsewhere)**

MTP: Week 4 Meal Planner

Day	1st Meal Hour	2nd Meal Hour
Monday		
Tuesday		
Wednesday		
Thursday		
Friday		
Saturday		
Sunday		

To simplify things, make extra serves for the following day.

MTP: Week 4 Shopping List

“Keto” Fruits (no fleshy fruits!)<br>(berries, avocados, olives etc)	Vegetables<br>(cauliflower, broccoli, capsicum etc)
Leafy Greens<br>(spinach, kale, lettuce etc)	Proteins<br>(meats, eggs, cheese etc)
Fats<br>(extra virgin olive oil, butter etc)	Pantry<br>(almond meal, ground flaxseed etc)
Nuts and Seeds<br>(almonds, walnuts, chia seeds etc)	Other<br>(whatever doesn’t fit elsewhere)
